# Systematic review and meta-analysis of neurofeedback and its effect on posttraumatic stress disorder

**DOI:** 10.3389/fpsyt.2024.1323485

**Published:** 2024-03-21

**Authors:** Jeffrey D. Voigt, Michael Mosier, Aron Tendler

**Affiliations:** ^1^ Medical Device Consultants of Ridgewood, LLC, Waldwick, NJ, United States; ^2^ EMB Statistical Solutions, LLC, Topeka, KS, United States; ^3^ Department Life Sciences, Ben-Gurion University of the Negev, Be’er Sheva, Israel

**Keywords:** neurofeedback, PTSD, systematic review, meta-analysis, randomized trial

## Abstract

**Background:**

To date, only one systematic review and meta-analysis of randomized controlled trials (RCTs) has evaluated the effect of neurofeedback in PTSD, which included only four studies and found an uncertainty of the effect of EEG-NF on PTSD symptoms. This meta-analysis is an update considering that numerous studies have since been published. Additionally, more recent studies have included fMRI-NF as well as fMRI-guided or -inspired EEG NF

**Methods:**

Systematic literature searches for RCTs were conducted in three online databases. Additional hand searches of each study identified and of systematic reviews and meta-analyses published were also undertaken. Outcomes evaluated the effect of neurofeedback vs. a control (active, sham, and waiting list) on their effects in reducing PTSD symptoms using various health instruments. Meta-analytical methods used were inverse variance random-effects models measuring both mean and standardized mean differences. Quality and certainty of the evidence were assessed using GRADE. Adverse events were also evaluated.

**Results:**

A total of 17 studies were identified evaluating a total of 628 patients. There were 10 studies used in the meta-analysis. Results from all studies identified favored neurofeedback’s effect on reducing PTSD symptoms including BDI pretest–posttest [mean difference (MD): 8.30 (95% CI: 3.09 to 13.52; P = 0.002; *I* ^2^ = 0%)]; BDI pretest–follow-up (MD: 8.75 (95% CI: 3.53 to 13.97; P < 0.00001; *I* ^2^ = 0%); CAPS-5 pretest–posttest [MD: 7.01 (95% CI: 1.36 to 12.66; P = 0.02; *I* ^2^ = 86%)]; CAPS-5 pretest–follow-up (MD: 10 (95% CI: 1.29 to 21.29; P = 0.006; *I* ^2^ = 77%); PCL-5 pretest–posttest (MD: 7.14 (95% CI: 3.08 to 11.2; P = 0.0006; *I* ^2^ = 0%); PCL-5 pretest–follow-up (MD: 14.95 (95% CI: 7.95 to 21.96; P < 0.0001; *I* ^2^ = 0%). Other studies reported improvements using various other instruments. GRADE assessments of CAPS, PCL, and BDI demonstrated a moderate/high level in the quality of the evidence that NF has a positive clinical effect.

**Conclusion:**

Based on newer published studies and the outcomes measured, NF has demonstrated a clinically meaningful effect size, with an increased effect size at follow-up. This clinically meaningful effect appears to be driven by newer fMRI-guided NF and deeper brain derivates of it.

## Introduction

Neurofeedback (NF) technologies in the treatment of posttraumatic stress disorder (PTSD) have evolved over the years. Prior systematic reviews and meta-analyses on randomized controlled trials (RCTs) have shown promising results using electroencephalogram (EEG) NF for PTSD but used traditional EEG NF technologies and with very small numbers of patients (1). More recent systematic reviews and meta-analyses have included duplicate studies (2) or non-randomized studies (2, 3) or been incomplete in their systematic review of RCTs (4, 5). Additionally, several RCTS (using newer forms of deep brain feedback—functional magnetic resonance imaging [fMRI] NF and fMRI informed EEG NF) have been published in the past 2–3 years.

The neuroscientific rationale in using NF in treating PTSD has been studied extensively. At its essence, NF appears to strengthen or rebalance the brain’s network and a patient’s regulatory capacity (6). In patients who have PTSD, those portions of the brain which control emotional regulation (prefrontal cortex, hippocampus, and amygdala) work less effectively—with the prefrontal cortex and hippocampus decreasing in volume (6). The amygdala, however, becomes much more active in PTSD patients (6, 7). Real-time functional magnetic resonance imaging (rt-fMRI) can target these deeper brain regions, evaluate their activity, and evaluate effects of such therapies as NF (8–12) using accepted PTSD instruments such as the clinician-administered PTSD scale (CAPS-5) and the PTSD Checklist for DSM-5 (PCL-5). Early studies on the use of NF EEG with fMRI have demonstrated an effect on these areas of the brain (13).

Neurofeedback technologies have recently been developed which fuse simultaneous EEG and fMRI recordings of the amygdala to produce a statistical model (referred to as electronic fingerprint [EFP]–EEG-fMRI-pattern) biomarkers which can measure the effect of EEG NF training on healthy individuals and PTSD patients’ response to traumatic/nontraumatic stimuli (14, 15). The Food and Drug Administration (FDA) cleared in early 2023 indications for use of one such amygdala-EEG-NF therapy for use in conjunction with evidence-based treatments for PTSD such as psychotherapy and pharmacotherapies (16, 17). Unfortunately, none of the newer deeper brain NF RCT studies have been included in a systematic review and meta-analysis.

The purpose of this analysis is to provide an updated systematic review and meta-analysis on the use of NF in the treatment of PTSD.

## Methods

Eligibility criteria for the systematic review included confirmed diagnosis of PTSD (with the possibility of comorbid conditions); the use of neurofeedback based on EEG only or guided/inspired *a priori* by fMRI and associated algorithms (i.e., EFP); randomized controlled trial, with or without adjunctive treatments such as pharmacotherapy or psychotherapy; and any age group. Exclusion criteria included traumatic brain injury, active psychosis, personality disorder, active suicidal ideation, pregnancy, schizophrenia, and major neurological disorder. For the purposes of the analysis, the following definitions were used for NF: NF which incorporates all types of NF including EEG, fMRI, and fMRI-informed EEG; EEG NF: refers to the use of EEG in providing NF; fMRI NF: utilizes fMRI in providing NF; and fMRI-informed EEG-NF: utilizes an EEG activity pattern which is a surrogate of fMRI in providing the NF.

The systematic review was conducted using the electronic databases PubMed Central, Cochrane CENTRAL, and EBSCO/CINAHL and used the following search terms: [(neurofeedback AND random*) AND trial] AND PTSD [Note: Cochrane Central search did not use the term random]. All searches were performed on 05/10/2023 and updated on 19/12/2023. All databases were searched from 01/01/1990 to 19/12/2023. Reference lists of relevant articles (e.g., prior systematic reviews and/or meta-analyses on the use of NF and PTSD) were hand searched for additional references. Additionally, ClinicalTrials.gov was searched to compare studies that were listed as completed with publications of those studies. One of the authors selected the relevant studies from the searches, and the other authors reviewed and commented on the selections. If there was disagreement on the articles selected, it was resolved by consensus along with the use of the Cochrane methodology (18). Excluded studies and the reasons for their exclusion are provided in **Supplementary Data Sheet 1**.

### Data collection and evaluation

One author extracted the data (JV) on study characteristics and outcomes, and the other authors reviewed and verified the extracted data. Data were extracted on study design and methods, participants (including baseline characteristics, inclusion and exclusion criteria), interventions (experimental and control and their components), outcomes assessed (including how they were measured and their duration), and any competing interests and follow-up required to the authors of the published papers. A Preferred Reporting Items for Systematic Reviews and Meta-Analyses (PRISMA) checklist was used to identify all relevant components of a systematic review and meta-analysis (19) (**Supplementary Data Sheet 2**). Lastly, **Supplementary Data Sheet 3** shows the Consensus on the reporting and experimental design of cognitive behavioral neurofeedback studies (CRED-nf checklist) for each individual study.

### Assessment of risk of bias and certainty of evidence

Risk of bias was assessed for each study and certainty of evidence related to outcome level. All authors critically appraised the included studies following the Cochrane risk of bias tool which assesses the following: selection bias (randomization and randomization sequence, allocation concealment), performance bias (blinding of patients, clinicians), detection bias (those assessing outcomes), attrition bias (amount, nature, or handling of incomplete data), reporting bias (selective outcome reporting), and any conflicts of interest (by clinicians, including funding of/involvement with studies by manufacturers). As mentioned above, ClinicalTrials.gov was assessed with the purpose of comparing completed studies with publications of those studies (publication bias).

For certainty of evidence, the Grading of Recommendations Assessment, Development and Evaluation (GRADE) was used, which is a sequential process for preparing evidence profiles (summaries) and developing evidence-based recommendations after a thorough review and assessment of evidence. GRADE is an approach for assessing the overall certainty of the evidence (e.g., how confident one can be in making evidence-based medical decisions). Those meta-analyses which included at least five studies were assessed with GRADE.

Initial scoring was performed by the lead author related to Cochrane assessments and on the CRED-nf checklist. These scores were then forwarded on to the other authors for their assessments. Scores provided were based on the majority interpretation.

### Data synthesis and analysis

The results of each study were summarized, and risk of bias was assessed, as shown in **Supplementary Data Sheet 1**. Where possible, data were combined by outcome in meta-analysis to better understand the aggregated effect (if three or more studies were involved). Both mean differences (using the same health instrument) and standardized mean differences (Hedges’ g) were calculated using inverse variance random-effects models with 95% confidence intervals (CIs). Effect sizes reported are based on the standardized mean differences. Heterogeneity was assessed using *I^2^* statistics. Meta-analysis was performed using Review Manager (RevMan) Version: 5.4.1, The Cochrane Collaboration 2020. Funnel plots were to be used to investigate publication bias if the number of studies in a meta-analysis was at least 10 (18). Adverse events were quantified. For those meta-analysis where high heterogeneity existed (*I^2^* >60%) (18), a sensitivity analysis was undertaken to determine which of any of the studies included in the meta-analysis was affecting it and the reasons why. Data missing from studies were assumed to be missing at random. Outcomes evaluated were those listed as either primary or secondary in each of the studies with the main outcomes as follows: Clinician Administered PTSD Scale (CAPS-5), PTSD Checklist for DSM-5 (PCL-5), and Beck Depression Inventory (BDI). Lastly, as part of the analysis, we examined the impact on outcomes of active (e.g., other biofeedback, yoked) or passive controls (treatment as usual [TAU] or standard of care [psychotherapy, pharmacotherapy]; or waitlist) vs. NF, as it has been found that there are group differences between active and passive controls (20). This separate analysis was performed when there were ≥3 studies involved.

## Results

### Search results

**Figure 1:**
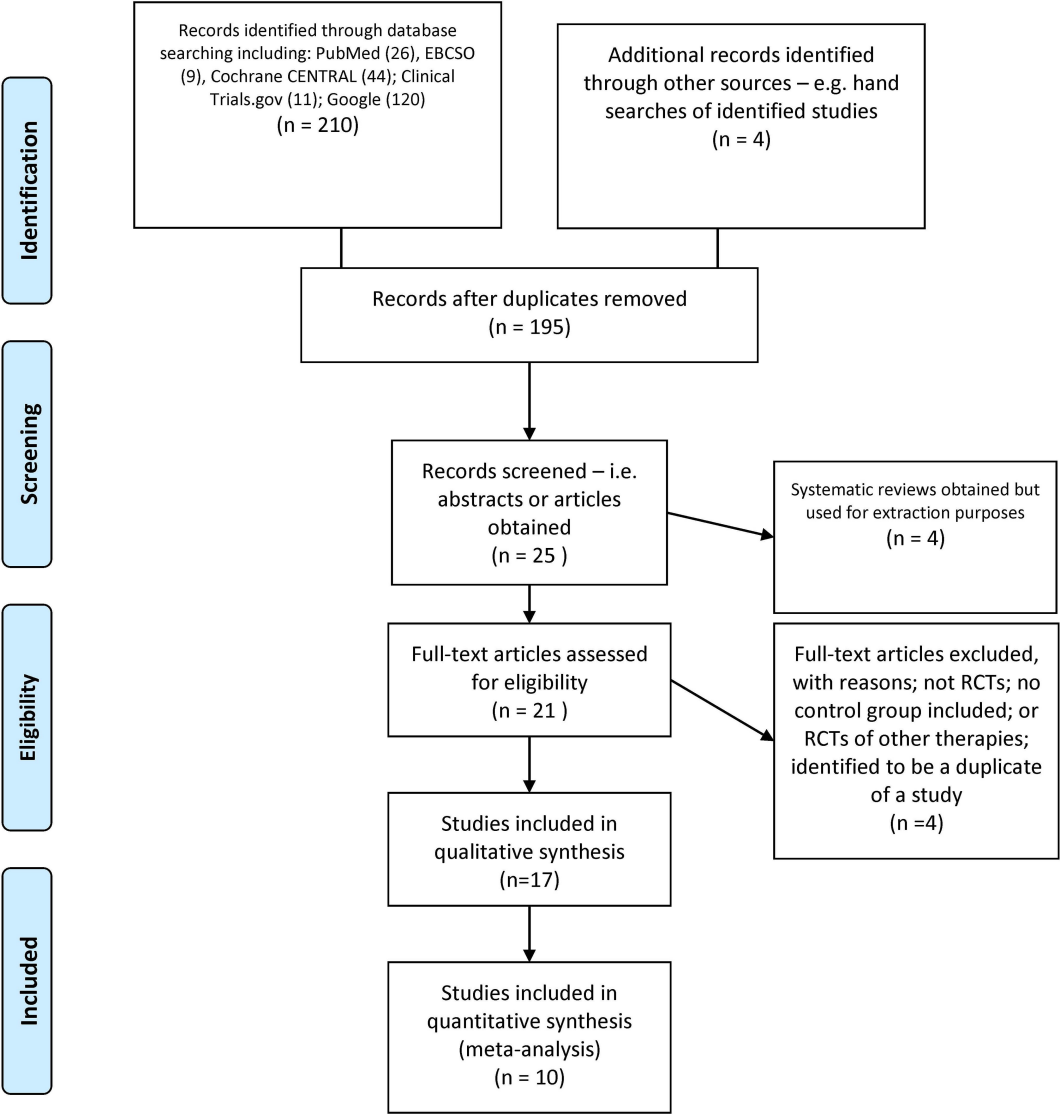
PRISMA flow diagram. Reproduced from Moher et al. (21), licensed under CC BY 4.0.

The literature search identified 195 records after duplicates were removed. In reviewing the abstracts, 170 articles were excluded due to lack of relevance. Systematic reviews with or without meta-analysis on PTSD treatments were excluded (4) but were evaluated for potential NF RCTS. Of the 25 full-text articles obtained, four were reviews and were evaluated for identification of NF RCTS and four were excluded due to not being RCTs, RCTs with no control group, or RCTs of other therapies (these eight were excluded). This left 17 studies for qualitative synthesis of which 10 were used for meta-analytic purposes (**Figure 1**). **Supplementary Data Sheet 4** shows the search strategy and disposition of articles identified.

### Characteristics of included studies

After duplicates were removed (21–24), 17 randomized controlled trials, involving 628 patients (55% men), met the inclusion criteria and were included in the review (16, 25–40). The studies took place from 1991 to 2023, with eight in the United States (25–28, 30, 31, 34, 35), one in Canada (32), two in Iran (29, 36), two in Israel (16, 38), and one each in Germany (37), South Korea (33), the Netherlands (39), and Nepal (40). All but three of the studies were on adults (31, 39, 40). Of the 17 studies, three compared NF with yoked feedback (27, 32, 34), four compared NF with waitlist (the waitlist group received the same NF after the trial was completed) (28, 31, 33, 40), five compared NF with the standard of care/treatment as usual the patient was presently on (16, 25, 36, 38, 39), two compared NF with no treatment (26, 29), one compared NF with a form of biofeedback (HRV biofeedback) (35), one compared NF with a form of relaxation (37), and one compared NF with sham neurofeedback (30).

There were 13 of 17 studies that used EGG NF (25–29, 31–33, 35–37, 39, 40). Two studies used fMRI NF (30, 34), and two studies utilized fMRI informed EEG-NF (16, 38). Additionally, one study used fMRI scans as an assessment tool for the mechanism of the NF training (32).

Treatment duration averaged 8.2 ± 5.1 weeks (range: 3–20 weeks), and the number of total sessions averaged 17.2 ± 7.7 sessions (range: 3–28 sessions). Outcome effect was measured at the end of therapy in eight studies (25–27, 30, 35–37, 39), and the other nine studies measured outcome effect at the end of therapy and 1–6 months after the end of the therapy. Studies ranged in enrollment size from 10 to 77 patients (average 36.9 ± 15.3). The included studies and their characteristics as outlined above are presented in **Table 1**.

**Table 1:** Study characteristics.

Study	NF	Control	Total	Treatment modality	No. of females	Country	Control	Treatment duration (wks)	No. of sessions	Outcome and follow-up	Attrition NF
van der Kolk 2016 (28)	28	24	52	EEG NF	40	USA	Waitlist	12	24	End therapy/1 mth post	6
Kelson 2013 (26)	5	5	10	EEG NF	0	USA	No treatment	4	20	End therapy	2
Leem 2021 (33)	10	9	19	EEG NF	17	South Korea	Waitlist	8	16	End therapy/1 mth post	1
Noohi 2017 (29)	15	15	30	EEG NF	0	Iran	No treatment	6	25	End therapy/1.5 mts post	0
Onton 2016 (27)	36	36	72	EEG NF	5	USA	Yoked neurofeedback	4	Not reported	End therapy	12
Peniston 1991 (25)	15	14	29	EEG NF	0	USA	Standard of care	4	28	End therapy	0
Rogel 2020 (31)	20	17	37	EEG NF	13	USA	Waitlist	12	24	End therapy/1 mth post	5
Fruchtman-Steinbok 2021 (16)	25	13	38	amygdala EFP NF (fMRI informed EEG NF)	15	Israel	Standard of care	13	15	End therapy/3 and 6 mth post	12
Nicholson 2020 (32)/Shaw 2023 (21)/Nicholson 2023 (24)	20	18	38	fMRI separately after each alpha-rhythm EEG NF (fMRI informed EEG NF)	27	Canada	Yoked neurofeedback	20	20	End therapy/3 mth post	0
Zhao 2023 (34)	14	11	25	real-time fMRI amygdala NF at baseline and end of treatment	21	USA	Yoked neurofeedback	3	3	End therapy/1 and 2 mth post	0
Zotev 2018 (22)/Misaki 2018 (30)/Misaki 2021 (23)	25	11	36	real-time fMRI amygdala NF at baseline and end of treatment	0	USA	Sham neurofeedback	11.5	7	End therapy	3
Bell 2019 (35)	12	11	23	Loreta z-score NF - EEG cap and 3D source imaging (for targeted, real-time training of deeper areas of brain)	unknown	USA	Biofeedback	7	15	End therapy	1
Yeganeh 2015 (36)	15	15	30	EEG NF	0	Iran	Standard of care	Not reported	20	End therapy	0
Winkeler 2022 (37)	18	18	36	EEG NF	36	Germany	Media-supported relaxation without EEG	6	12	End therapy	0
Fine 2023 (38)	40	15	55	amygdala EFP NF (fMRI informed EEG NF)	55	Israel	Psychotherapy (TAU)	8	10	End therapy, 1, 3, and 6 months post therapy	1
Schuurmans 2021 (39)	37	40	77	EEG NF	31	Netherlands	TAU	6	12	End of therapy	4
Antle 2018 (40)	9	12	21	EEG NF	21	Nepal	Waitlist	6	24	End of therapy, 2 months post therapy	0
Total	344	284	628		280		Mean	8.2	17.2		48
				percent Fem.	44.6%		Std dev	5.1	7.73	Attrition	7.6%

Amygdala EFP NF, amygdala electronic fingerprint neurofeedback; EEG NF, electroencephalogram neurofeedback; fMRI, functional magnetic resonance imaging; TAU, treatment as usual

**Figure 2:**
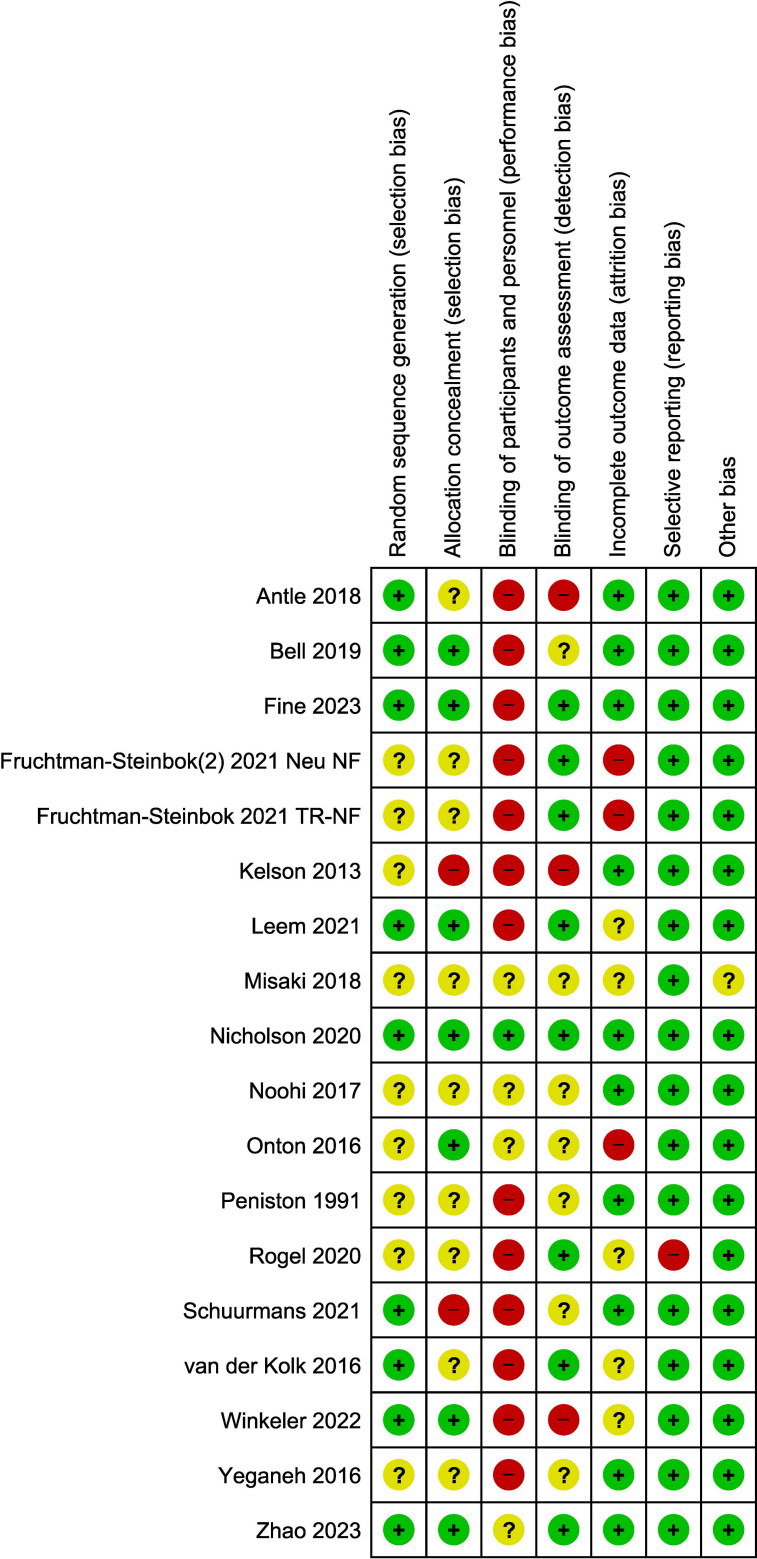
Risk of bias summary.

**Figure 3:**
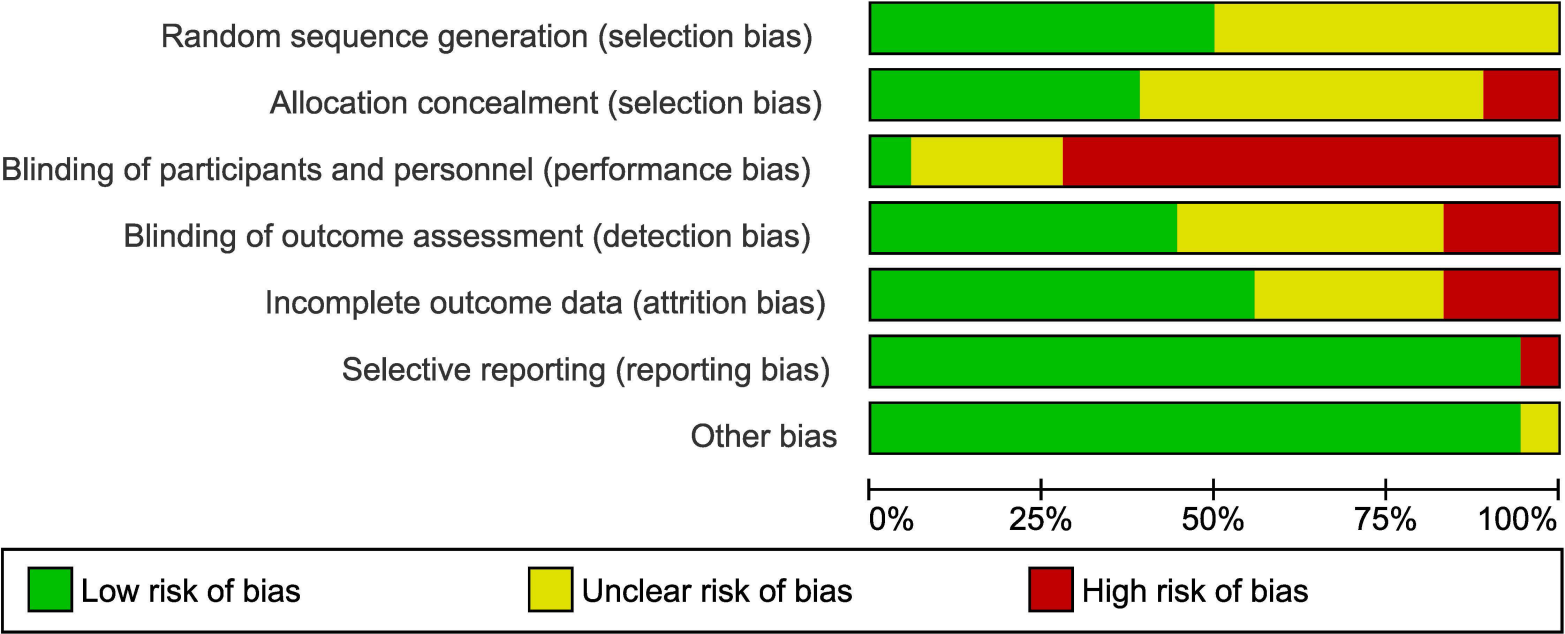
Risk of bias graph.

Studies were assessed as mainly having a low to moderate (unclear) risk of bias. The main issue with bias had to do with a lack of blinding of patients and/or clinical personnel administering the therapy (13 out of 17 studies; 76% of the studies). However, blinding of the outcome assessment was performed in seven of these 17 studies (low risk). The assessment of risk-of-bias graph and summary are presented in **Figures 2** and **3**, respectively. As it relates to the attrition bias, 7.6% of those randomized dropped out of the trial at some point during the trial (48 out of 628).

As it relates to an assessment using the CRED-nf best practices checklist, **Supplementary Data Sheet 3** shows which domains were included in the studies included in this analysis. In general, the more recent published studies (from 2021 to the present) included more of these domains than studies published prior to 2021 (67% vs. 53%)—with this increase likely being due to the introduction of the CRED-nf checklist in 2020. The best practices not addressed in a large portion of the studies included justification of sample size, blinding of patient and clinician administering the NF/control, a lack of reporting on and justifying the reinforcement schedule used for NF, and the plotting of with-in and between session feedback variables.

### Outcomes

Effects of NF on PTSD symptoms were assessed *via* meta-analyses in 10 of the 17 studies (16, 28–30, 32–36, 38,).

#### CAPS-5 pre- and post-therapy assessment (N = 221 patients)

**Figure 4:**
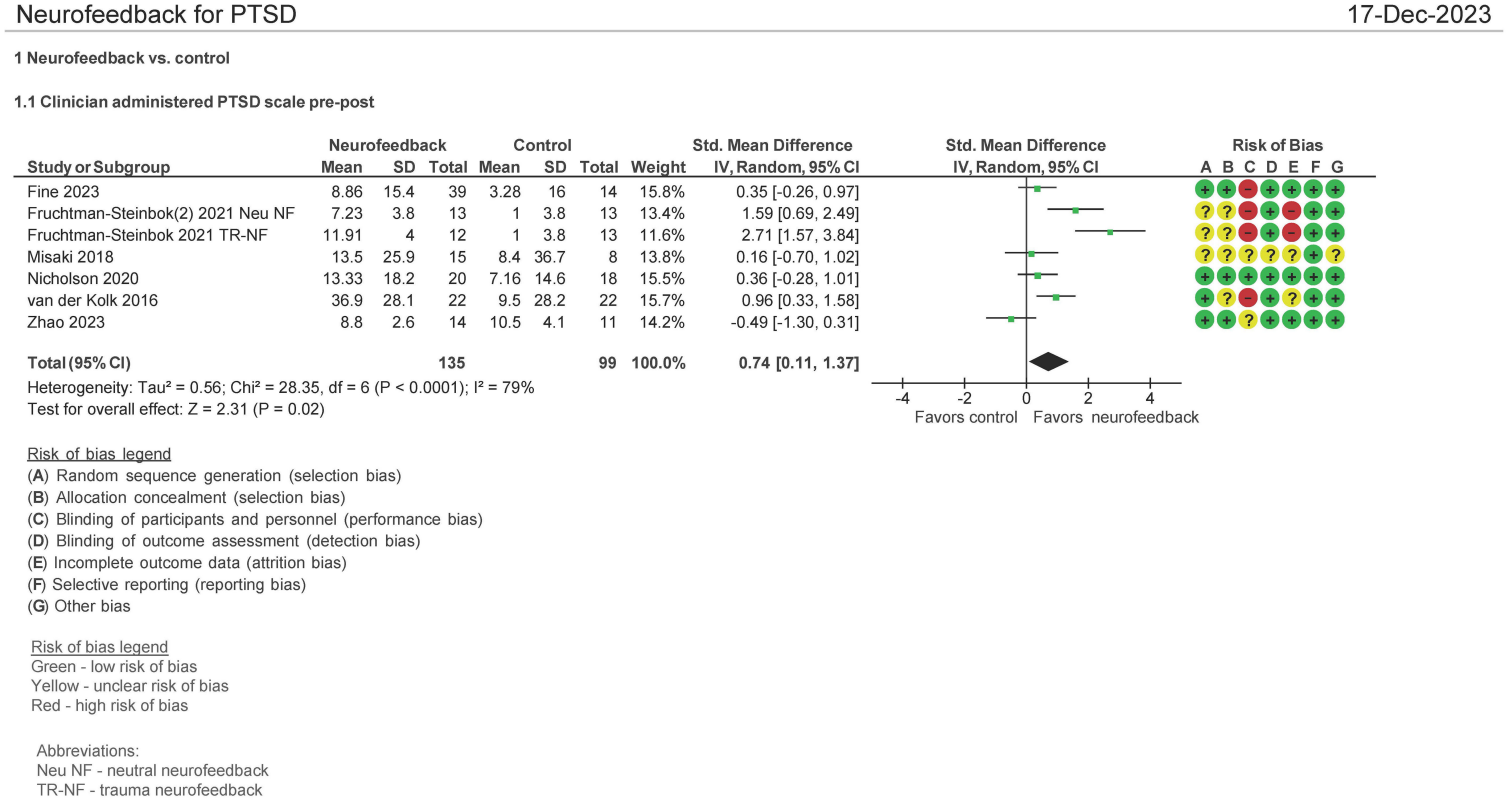
Clinician Administered PTSD Scale (CAPS-5) pre and post treatment.

Seven studies evaluated the effect of the use of NF in PTSD using the Clinician Administered PTSD scale (CAPS) pre and post therapy (16, 28, 30, 32, 34, 38). Both mean differences and standardized mean differences were calculated using inverse variance random-effects models with 95% confidence intervals (CIs). The mean difference (MD) was 7.01 (95% CI: 1.36 to 12.66; P = 0.02; *I* ^2^ = 86%), and the standardized mean difference (SMD) was 0.74 (95% CI: 0.11 to 1.37; P = 0.02; *I* ^2^ = 79%) (**Figure 4**). Both meta-analyses favored NF.

The patients treated with NF in this meta-analysis cohort (N = 135) were diagnosed as having mild (16, 32, 34, 38), moderate (30), and severe (28) PTSD. 44% of them were on concomitant pharmacotherapy and 64% on psychotherapy. One study identified patients as having chronic PTSD (28). 30% of patients were men, and the age ranges were on average 30–46 years. 74% of the patients’ cause of PTSD symptoms originated from physical, sexual, and neglect. **Table 2A** (neurofeedback) and **2B** (control) show the baseline characteristics of the patients.

**Table 2A:** CAPS-5 pre–posttreatment—neurofeedback group.

Baseline characteristics	Fruchtman 2021 (16) (neutral NF)	Fruchtman 2021 (16) (trauma NF)	Misaki 2018 (30)	Nicholson 2020 (32)	Van der Kolk 2016 (28)	Zhao 2023 (34)	Fine 2023 (38)	Totals
N	13	12	15	20	22	14	40	136
Age	37.7 (10.7)	40.25 (21.96)	30.8 (5.4)	39.2 (12.08)	46.04 (12.89)	40.2 (14.27)	37.37 (11.45)	
Male/female	8/5	5/7	15/0	7/13	3/19	3/11	0/40	41/95
PTSD chronicity					22	14	40	
Medications (N on meds)	13	12	0	12	16	7		60
Medications (duration)								
Medications (type)				antidepressants (19); antipsychotics (6); sedatives (8); stimulants (2)	SSRI (7); stimulants (4); antipsychotics (3); bupropion (3); benzodiazepine (5)	antidepressants (7); stimulants (1)		
% on meds	100.0%	100.0%	0.0%	60.0%	72.7%	50.0%		44.1%
Psychotherapy (N)	13	12			22		40	87
% Psychotherapy	100.0%	100.0%	0.0%	0.0%	100.0%	0.0%		64%
Psychotherapy (duration mths)				N/A	6			
Psychotherapy (type)				N/A	trauma focused			
Country of origin	Israel	Israel	US	Canada	US	US	Israel	
Comorbidities (current)			MDD (8) alcohol dependency (2)	MDD (6)		MDD (5); OCD (3); social phobia (5)		
Comorbidities (past)			MDD (7)	MDD (9)		MDD (2)		
PTSD origin								
Military	3	1	15	4				23
First responder			0	2				2
Civilian (physical/sex/neglect)	12	11	0	14	22	14	40	101
CAPS baseline	37.84 (2.56)	32.83 (2.67)	51.7 (16.7)	36.52 (9.71)	80.98 (17.55)	33.71 (7.99)	40.52 (9.92)	
CAPS post	30.61 (2.87)	20.91 (2.99)	38.2 (19.8)	23.19 (15.37)	44.12 (22)	24.9 (13.9)	31.66 (11.83)	
Number NF sessions	15	15	7	20	24	3	10	13.43
NF duration (weeks)	13	13	11.5	20	12	3	8	11.5

CAPS, clinician administered PTSD scale; MDD, major depressive disorder; NF, neurofeedback; OCD, obsessive compulsive disorder; SSRI, selective serotonin reuptake inhibitors.

**Table 2B:** CAPS-5 pre–posttreatment—control group.

Baseline characteristics	Fruchtman 2021 (16)	Misaki 2018 (30)	Nicholson 2020 (32)	Van der Kolk 2016 (28)	Zhao 2023 (34)	Fine 2023 (38)	Totals
N	13	11	18	24	11	15	92
Age	32 (8.66)	34.1 (8.5)	46.28 (12.37)	42.45 (13.50)	50.36 (1278)	35.86 (9.43)	
Male/female	8/5	11/0	4/14	5/17	1/10	0/15	29/61
PTSD chronicity					11	15	26
Medications (N on meds)	13	0	12	10	4	0	39
Medications (duration)							
Medications (type)				SSRIs (6) Benzodiazepine (3); antianxiety (2); bupropion (2);SSNRI (2); tricyclic antidepressant (1)	Antidepressants (2); anticonvulsants (2)		
% on meds	100%	0%	67%	41.7%			42.4%
Psychotherapy (N)	13	0		24		15	52
% Psychotherapy	100%	0%		100%		100%	57%
Psychotherapy (duration mths)				6		12	
Psychotherapy (type)				Trauma focused			
Country of origin	Israel	US	Canada	US	US	Israel	
Comorbidities (current)		MDD (2)	MDD (7); somatization disorder (3); specific phobia (1)		MDD (5) social phobia (3)		
Comorbidities (past)			MDD (5)		MDD (2)		
PTSD origin							
Military	2	11	3				16
First responder			1				1
Civilian (physical/sex/neglect)	11		14	24	11	15	
CAPS baseline	37.93 (2.56)	57 (25.3)	39.94 (7.83)	76.24 95% CI (69.13, 83.86)	39.7 (9.3)	43.06 (10)	
CAPS post	36.92 (2.87)	53.8 (23.9)	32.78 (12.27)	66.49 95% CI (57.39, 75.6)	29.2 (15.4)		

CAPS, clinician-administered PTSD scale; MDD, major depressive disorder; NF, neurofeedback; OCD, obsessive compulsive disorder; SSRI, selective serotonin reuptake inhibitors.

In examining the heterogeneity in CAPS-5 pre and post therapy assessment, when Fruchtman-Steinbok (16) was removed from the meta-analysis, heterogeneity was reduced from 86% to 9%. The effect sizes of Fruchtman-Steinbok in both treatment arms of the study when compared with control were 1.59 (Neutral NF) and 2.71 (Trauma-NF). After removal of this study, the standardized mean difference (SMD) was also reduced to 0.15 (95% CI: −0.22 to 0.53; P = 0.42; *I* ^2^ = 9%).

In a separate meta-analysis examining studies comparing NF vs. passive controls (16, 28, 38) (waitlist, TAU, e.g., psychotherapy, no treatment; N = 135 patients) on the CAPS-5 endpoint, it was found that the SMD was 1.30 (95% CI: 0.44 to 1.27; P = 0.003; *I* ^2^ = 79%). Additionally, when examining NF vs. active controls (30, 32, 34) (yoked NF, sham; N = 86 patients), it was found that the SMD was 0.05 (95% CI: −0.47 to 0.56; P = 0.86; *I* ^2^ = 27%). However, this included a study where only three NF sessions were employed (34) (figure not shown).

#### CAPS-5 pre- and follow-up therapy assessment (N = 103 patients)

**Figure 5:**
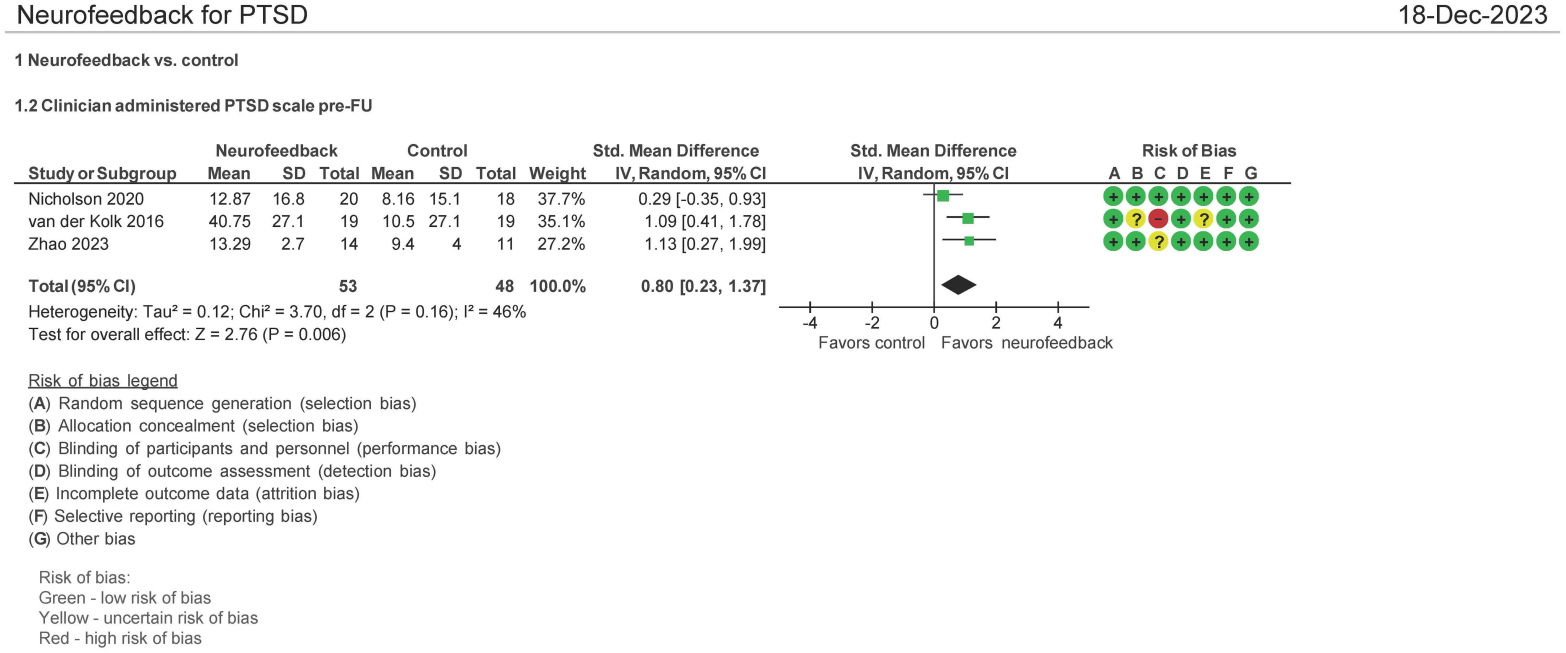
Clinician Administered PTSD Scale (CAPS-5) pre and follow-up treatment (1-3 months).

Three (3) studies evaluated the effect of the use of NF in PTSD using CAPS-5 pre and post therapy follow-up (1–3 months post therapy follow-up) (28, 32, 34). Both mean differences and standardized mean differences were calculated using inverse variance random-effects models with 95% confidence intervals (CIs). The MD was 10.00 (95% CI: 1.29 to 21.29; P = 0.006; *I* ^2^ = 77%), and the SMD was 0.80 (95% CI: 0.23 to 1.37; P = 0.006; *I* ^2^ = 46%) (**Figure 5**). Both meta-analyses favored NF.

The patients treated with NF in this meta-analysis cohort (N = 54) were diagnosed as having mild (32, 34) and severe PTSD (28). Of these patients, 62.5% were on concomitant pharmacotherapy and 39% on psychotherapy. One study identified patients as having chronic PTSD (28). 25% of patients were men, and the average age range was from 40 to 46 years. 46% of the patients’ cause of PTSD symptoms originated from the military with 50% originating from physical, sexual, and neglect. **Tables 3A** (NF) and **3B** (control) show the baseline characteristics of the patients.

**Table 3A:** CAPS pre-FU characteristics (NF group).

Baseline characteristics	Nicholson 2020 (32)	Van der Kolk 2016 (28)	Zhao 2023 (34)	Totals
N	20	22	14	56
Age	39.2 (12.08)	46.04 (12.89)	40.2 (14.27)	
Male/female	7/13	3/19	3/11	13/53
PTSD chronicity		22		
Medications (N on meds)	12	16	7	35
Medications (duration)				
Medications (type)	antidepressants (19); antipsychotics (6); sedatives (8); stimulants (2)	SSRI (7); stimulants (4); antipsychotics (3); bupropion (3); benzodiazepine (5)	Antidepressants (7); stimulants (1)	
% on meds	60%	72.7%	50.0%	65%
Psychotherapy (N)	0	22		22
% Psychotherapy				39.3%
Psychotherapy (duration mths)		6		
Psychotherapy (type)				
Country of origin	Canada	US	US	
Comorbidities (current)	MDD (6)		MDD (5); OCD (3); social phobia (5)	
Comorbidities (past)	MDD (9)		MDD (2)	
PTSD origin				
Military	4	22		26
First responder	2			2
Civilian (physical/sex/neglect)	14		14	28
CAPS baseline	36.52 (9.71)	80.98 (17.55)	33.71 (7.99)	
CAPS FU	23.65 (13.71)	40.23 (18.4)	20.42	
Number NF sessions	20	24	3	15.67
NF duration (weeks)	20	12	3	11.67

CAPS, clinician-administered PTSD scale; MDD, major depressive disorder; NF, neurofeedback OCD, obsessive compulsive disorder; SSRI, selective serotonin reuptake inhibitors.

**Table 3B:** CAPS pre-FU characteristics (control group).

Baseline characteristics	Nicholson 2020 (32)	Van der Kolk 2016 (28)	Zhao 2023 (34)	Totals
N	18	24	11	53
Age	46.28 (12.37)	42.45 (13.50)	50.36 (1278)	
Male/female	4/14	5/17	1/10	10/41
PTSD chronicity			11	11
Medications (N on meds)	12	10	4	26
Medications (duration)				
Medications (type)		SSRIs (6) Benzodiazepine (3); antianxiety (2); bupropion (2);SSNRI (2); tricyclic antidepressant (1)	Antidepressants (2); anticonvulsants (2)	
% On meds	67%	41.7%		49%
Psychotherapy (N)		24		24
% Psychotherapy		100%		45%
Psychotherapy (duration mths)		6		
Psychotherapy (type)		Trauma focused		
Country of origin	Canada	US	US	
Comorbidities (current)	MDD (7); somatization disorder (3); specific phobia (1)		MDD (5) social phobia (3)	
Comorbidities (past)	MDD (5)		MDD (2)	
PTSD origin				
Military	3			3
First responder	1			1
Civilian (physical/sex/neglect)	14	24	11	49
CAPS baseline	39.94 (7.83)	76.24 95% CI (69.13, 83.86)	39.7 (9.3)	
CAPS post	32.78 (12.27)	66.49 95% CI (57.39, 75.6)	29.2 (15.4)	

CAPS, clinician-administered PTSD scale; MDD, major depressive disorder; NF, neurofeedback; OCD, obsessive compulsive disorder; SSRI, selective serotonin reuptake inhibitors.

#### PCL-5 pre and post therapy assessment (N = 166 patients)

**Figure 6:**
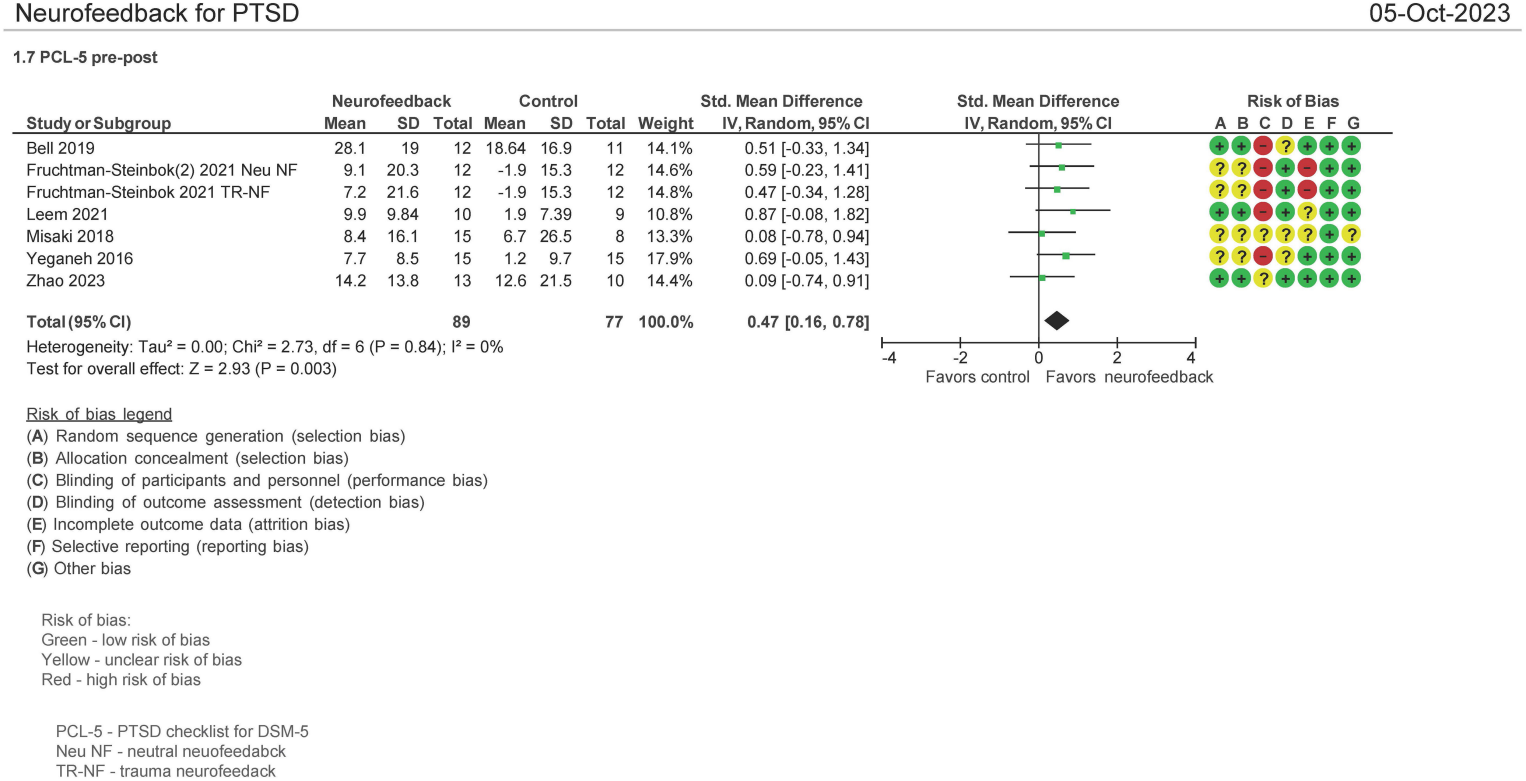
PTSD checklist for DSM-5 (PCL-5) pre and post treatment.

Seven (7) studies evaluated the effect on the use of NF in PSTD using the PTSD Checklist for DSM-5 (PCL-5) instrument pre and post therapy (16, 30, 33–36, 38). Both mean differences and standardized mean differences were calculated using inverse variance random-effects models with 95% confidence intervals (CIs). The MD was 7.14 (95% CI: 3.08 to 11.2; P = 0.0006; *I* ^2^ = 0%), and the SMD was 0.47 (95% CI: 0.16 to 0.78; P = 0.0003; *I* ^2^ = 0%) (**Figure 6**). Both meta-analyses favored NF.

The patients treated with NF in this meta-analysis cohort (N = 93) were diagnosed as having mild (30, 33–35), moderate (16), and severe PTSD (16, 36). Of these patients, 57% were on concomitant pharmacotherapy and 40% on psychotherapy. One study identified patients as having chronic PTSD (35). 69% of the patients were men, and the average age range was from 31 to 49 years. 51% of the patients’ cause of PTSD symptoms originated from physical, sexual, and neglect. **Tables 4A** (NF) and **4B** (control) show the baseline characteristics of the patients.

**Table 4A:** PCL-5 pre–post characteristics (NF group).

Baseline characteristics	Fruchtman 2021 (16) (neutral NF)	Fruchtman 2021 (16) (trauma NF)	Leem 2021 (33)	Misaki 2018 (30)	Zhao 2023 (34)	Bell 2019 (35)	Yeganeh 2016 (36)	Totals
N	15	12	10	15	14	12	15	93
Age	37.7 (10.7)	40.25 (21.96)	44.4 (13.6)	30.8 (5.4)	40.2 (14.27)	44.6 (13.1)	48.73	
Male/female	9/6	5/7	1/9	15/0	3/11		15/0	48/33
PTSD chronicity						12		12
Medications (N on meds)	15	12	10	0	7	9	15	68
Medications (duration)								
Medications (type)					antidepressants (7); stimulants (1)			
% On meds	100.0%	100.0%	100.0%	0.0%	50.0%	75.0%	100.0%	73.1%
Psychotherapy (N)	15	12	0			10		37
% Psychotherapy								39.8%
Psychotherapy (duration mths)								
Psychotherapy (type)								
Country of origin	Israel	Israel	South Korea	US	US	US	Iran	
Comorbidities (current)				MDD (8)	MDD (5); OCD (3); social phobia (5)			
Comorbidities (past)				MDD (7)	MDD (2)			
PTSD origin								
Military	3	1		15			30	49
First responder				0				
Civilian (physical/sex/neglect)	12	11	10	0	14			47
PCL baseline	61.4 (11.1)	53.75 (16.5)	44.3 (10.9)	42.2 (10.6)	43.43 (13.42)	46.2 (14.2)	68.7 (6.3)	
PCL post	52.3 (17.05)	46.6 (14)	34.4 (9.5)	36 (12.8)	29.23	18.1 (12.6)	60.9 (5.7)	
Number NF sessions	15	15	16	7	3	15	20	13
NF duration (weeks)	13	13	8	11.5	3	7		9.25

MDD, major depressive disorder; NF, neurofeedback; OCD, obsessive compulsive disorder; PCL, PTSD checklist for DSM-5.

**Table 4B:** PCL-5 pre–post characteristics (control).

Baseline characteristics	Fruchtman 2021 (16) (control)	Leem 2021 (33)	Misaki 2018 (30)	Zhao 2023 (34)	Bell 2019 (35)	Yeganeh 2016 (36)	Totals
N	13	10	15	14	12	15	93
Age	32 (8.66)	44.4 (13.6)	30.8 (5.4)	40.2 (14.27)	44.6 (13.1)	48.73	
Male/female	8/5	1/9	15/0	3/11		15/0	48/33
PTSD chronicity					12		12
Medications (N on meds)	13	10	0	7	9	15	68
Medications (duration)							
Medications (type)				Antidepressants (7); stimulants (1)			
% On meds	100%	100.0%	0.0%	50.0%	75.0%	100.0%	73.1%
Psychotherapy (N)	13	0			10		37
% Psychotherapy	100%						39.8%
Psychotherapy (duration mths)							
Psychotherapy (type)							
Country of origin	Israel	South Korea	USA	USA	USA	Iran	
Comorbidities (current)			MDD (8)	MDD (5); OCD (3); social phobia (5)			
Comorbidities (past)			MDD (7)	MDD (2)			
PTSD origin							
Military	2		15			30	47
First responder			0				
Civilian (physical/sex/neglect)	11	10	0	14			34
PCL baseline	37.93 (2.56)	44.3 (10.9)	42.2 (10.6)	43.43 (13.42)	46.2 (14.2)	68.7 (6.3)	
PCL post	36.92 (2.87)	34.4 (9.5)	36 (12.8)	29.23	18.1 (12.6)	60.9 (5.7)	

MDD, major depressive disorder; NF, neurofeedback; OCD, obsessive compulsive disorder; PCL, PTSD checklist for DSM-5.

In a separate meta-analysis examining studies comparing NF vs. passive controls (16, 33, 36) (waitlist, TAU, no treatment; N = 97 patients) on the PCL-5 endpoint, it was found that the SMD was 0.64 (95% CI: 0.23 to 1.05; P = 0.002; *I* ^2^ = 0%). Additionally, when examining NF vs. active controls (30, 35) (biofeedback, sham; N = 46 patients), it was found that the SMD was 0.30 (95% CI: −.0.30 to 0.90; P = 0.33; *I* ^2^ = 0%) (figure not shown).

#### PCL-5 pre and follow-up therapy assessment (N = 117 patients)

**Figure 7:**
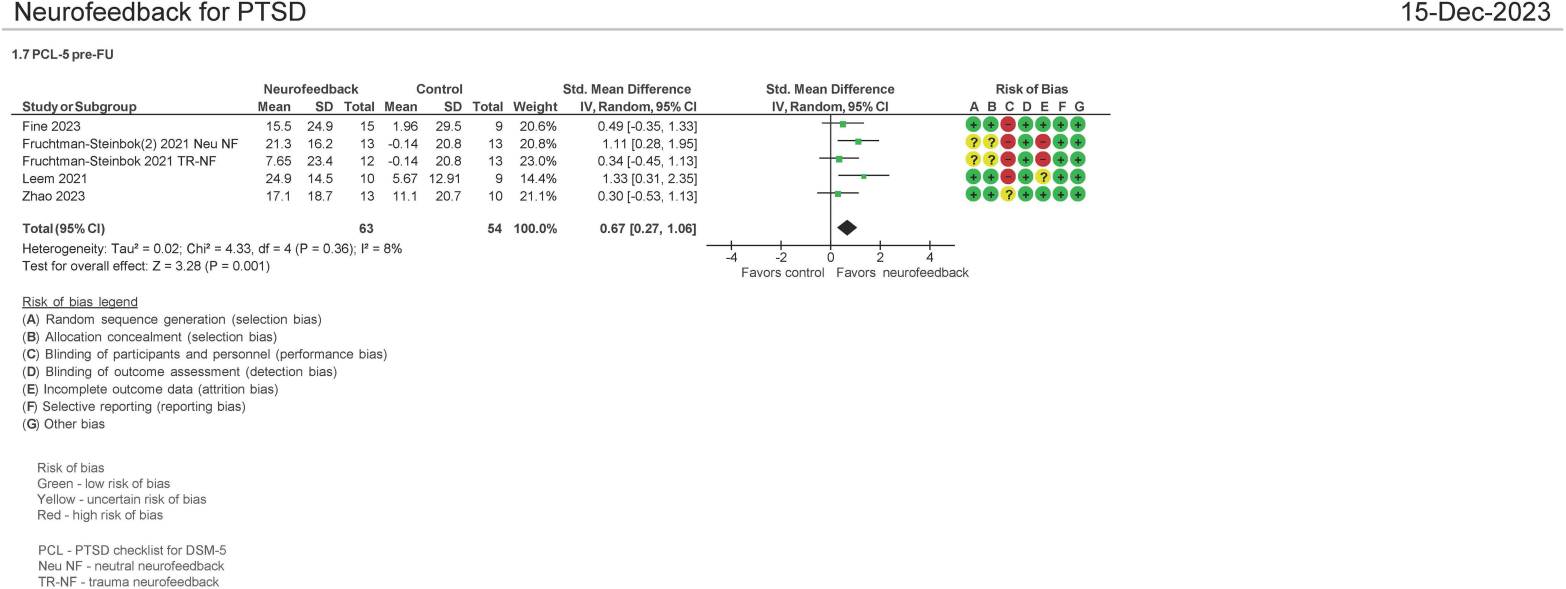
PTSD checklist for DSM-5 (PCL-5) pre and follow-up treatment (1-6 months).

Four studies evaluated the effect on the use of NF in PSTD using the PCL-5 instrument pre and FU therapy (1–6 months of follow-up) (16, 33, 34, 38). Both mean differences and standardized mean differences were calculated using inverse variance random-effects models with 95% confidence intervals (CIs). The MD was 14.95 (95% CI: 7.95 to 21.96; P < 0.0001; *I* ^2^ = 0%), and the SMD was 0.67 (95% CI: 0.27 to 1.06; P = 0.001; *I* ^2^ = 8%) (**Figure 7**). Both meta-analyses favored NF. A separate analysis was not undertaken comparing NF with passive controls and NF to active controls as three of the four studies examined passive controls (waitlist, psychotherapy, standard of care) (16, 33, 38).

### BDI pre and post therapy assessment (N = 95 patients)

**Figure 8:**
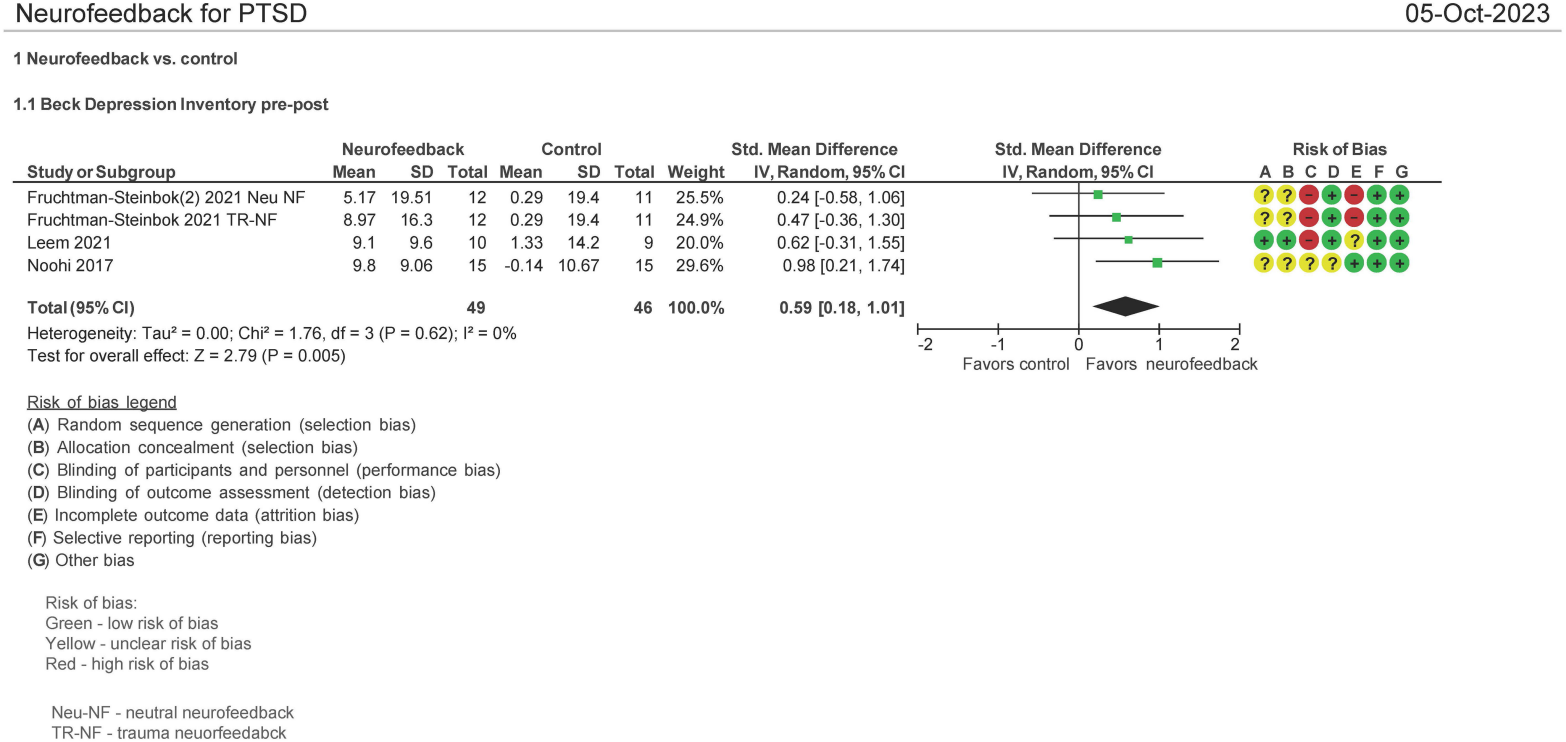
Beck Depression Inventory (BDI) pre and post treatment.

Three studies evaluated the effect on the use of NF in PSTD using the Beck Depression Inventory (BDI) pre and post therapy (16, 29, 33). Both mean differences and standardized mean differences were calculated using inverse variance random effects models with 95% confidence intervals (CIs). The MD was 8.75 (95% CI: 3.53 to 13.97; P = 0.001; *I* ^2^ = 0%), and SMD was 0.59 (95% CI: 0.18 to 1.01; P = 0.005; *I* ^2^ = 0%) (**Figure 8**). Both meta-analyses favored NF.

### Other outcomes not included in meta-analyses

#### BDI pre and follow-up therapy assessments

There were two studies which evaluated the BDI pre and follow-up (1–6 months of follow-up) (16, 33). Both studies demonstrated a statistically meaningful effect of NF vs. control on the BDI instrument.

#### MMPI and medication consumption pre and post therapy assessments

The Minnesota Multiphasic Personality Inventory was used to assess combat veterans (N = 29) with PTSD after EEG NF therapy (BWT) (25). BWT significantly reduced anxiety-provoking traumatic recurring nightmares/flashbacks and psychotropic medications for PTSD compared with traditional medical treatment.

#### CBCL, BRIEF, TSYYC, CAM, K-SADS, pre–posttreatment, and pre and 1-month follow-up posttreatment

In children 6–13 years of age with histories of severe abuse and neglect (PTSD) (N = 37), NF significantly decreased/improved upon PTSD symptoms, Child Behavior Checklist (CBCL) emotional and behavioral problems, Behavior Rating Inventory of Executive Function (BRIEF), Trauma Symptom Checklist for Young Children (TSCYC), Child Alexithymia Measure (CAM), and Kiddie Schedule for Affective Disorders and Schizophrenia for School Aged Children (K-SADS) at the end of 12 weeks of therapy vs. treatment as usual (31). At 1-month follow-up, there was no statistical difference between the groups (31).

#### Likert scale

Veterans with PTSD (N = 10) were evaluated on a Likert questionnaire (1–5 rating scale; 1—not at all; 5—extreme) regarding perceived PTSD symptom levels. EEG NF veterans vs. waitlist demonstrated a significant reduction in PTSD symptoms (ANOVA, P = 0.0004) after undergoing 20 NF sessions vs. waitlist (26).

#### IES-R, WCST, ToL pre–posttreatment, and pre and 1.5-month follow-up posttreatment

The Impact of Event Scale (IES) measuring PTSD symptoms, Wisconsin Card Sorting Test (WCST) (executive function), and Tower of London (ToL) [executive cognition] were not statistically different at the end of 6 weeks of EEG NF and at 1.5-month follow-up posttreatment vs. control (N = 29) in a trial completed in Iran (29). However, in a German study in patients being treated for comorbid chronic eating disorder and PTSD, it was found that the avoidance subscale (of cognitive or behavioral contact with the traumatic situation) was significantly lower posttreatment vs. pretreatment compared with the control group, which demonstrated no statistically significant difference pre- vs. posttreatment (37).

### STAI, TAS-20, and ERQ pre, post, and 3- and 6-month posttreatment

The State-Trait Anxiety Inventory (STAI), Toronto Alexithymia Scale (TAS-20), and Emotion Regulation Questionnaire (ERQ) were evaluated in one study using fMRI-informed EEG NF (16). Each of these scores improved at all evaluation time frames relative to no fMRI-informed EEG NF.

### Neurobiological assessments

One study examined adolescents’ response post-NF training (Muse, a game mediated intervention) plus TAU vs. TAU using measurements of their autonomic nervous system (ANS), hypothalamic–pituitary adrenal (HPA) hair cortisol (hC) levels, and HPA saliva cortisol (sC) levels in stress-related activities (39) in order to determine if the NF Muse training had an effect on them. Participants in the Muse intervention exhibited lower sympathetic nervous system (SNS) activity during rest and increased levels of sC response to acute stress. The lower SNS activity is helpful in reducing PTSD symptoms, stress, anxiety, depression, and aggression. Further stress reactivity in adolescents with PTSD symptoms exhibit blunted HPA reactivity to acute stress. The use of Muse helped to “restore” this blunted response to more normal levels.

### Other

In a study of girls 5–11 years of age with PTSD using an NF training (Mind-Full), it was demonstrated using a self-developed survey examining Mind-Full’s effect on calmness and attention in everyday life that Mind-Full demonstrated a significant effect on calming and a trend toward more attentive behavior vs. waitlisted children (40).

There was one ongoing clinical trial that is not included in this meta-analysis as the results could not be obtained and the abstract states the trial is ongoing (41).

#### Adverse events

Only four of the 17 studies included in this analysis reported on adverse events/complications. In three of these studies, there were no adverse events (33, 34) and one reported on self-injurious behavior in the control group (37).

#### Cost effectiveness

One study using EEG NF evaluated cost effectiveness using the EuroQol-5D as the quality of life instrument (33). It found that the incremental cost per quality-adjusted life year was $15,600. The study was performed in South Korea.

#### GRADE assessments

The quality of the evidence was rated as high regarding the CAPS-5 and PCL-5 posttreatment. For CAPS-5, PCL-5, and pre and post follow-up treatment, the quality of the evidence was also rated as high. For BDI pre–post, the quality of the evidence was rated moderate. GRADE assessments can be found in **Table 5**.

**Table 5:** Grading of Recommendations, Assessment, Development, and Evaluation (GRADE).

**Bibliography:** Misaki M, Phillips R, Zotev V, et al. Real-time fMRI neurofeedback positive emotional training normalized resting-state functional connectivity in combat veterans with and without PTSD: a connectome- side investigation. Neuroimage Clin. 2018;20:543-555. Nicholson A, Ros T, Densmore M, et al. A randomized, controlled trial of alpha-rhythm EEG neurofeedback in posttraumatic stress disorder: A preliminary investigation showing evidence of decreases PTSD symptoms and restored default mode and salience network connectivity using fMRI. Neuroimage 2020; 28:102490. Leem J, Cheong M, Lee H, et al. Effectiveness, cost-utility, and safety of neurofeedback self-regulating training in patients with post-traumatic stress disorder: A randomized controlled trial. Healthcare 2021;9:1351. Fruchtman-Steinbok T, Neynan j, Cohen A, et al. Amygdala electrical-finger print (AmygEFP) neurofeedback guided by individually-tailored trauma script for posttraumatic stress disorder: Proof of concept. Neuroimage. 2021;32:102859. Zhao Z, Duek O, Seidermann R, et al. Amygdala downregulation training using fMRI neurofeedback in post-traumatic stress disorder: a randomized, double-blind trial. Trans. Psych. 2023;13:177. Bell AN, Moss D, Kallmeyer RJ. Healing the neurophysiological roots of trauma: A controlled study examining LORETA Z-score neurofeedback and HRV biofeedback for chronic PTSD. Neuroreg. 2019;6(2):54- 70. Yeganeh ZA, et al. The effectiveness of neurofeedback training on reducing symptoms of war veterans with posttraumatic stress disorder. Prac Clin Psych. 2016;4(1):17-23. Fine NB, Helpman L, Armon DB, et al. Amygdala-related EEG neurofeedback as an add-on therapy for treatment resistant childhood sexual abuse PTSD: Feasibility study. Psych Clin Neuro. 2023; doi:10.1111/pcn.13591. Noohi S, Miraghaie A, Arabi A, Nooripour R. Effectiveness of neuro- feedback treatment with alpha/that method on PTSD symptoms and their executing function. Biomedical Research. 2017;28(5):2109-2027. Van der Kolk B, Hodgdon H, Gapen M, et al. A randomized controlled study of neurofeedback for chronic PTSD. PLOS ONE. 2016;11(12): e0166752.					
**Outcomes**	**No of Participants (studies)** Follow up	**Quality of the evidence Relative effect** (GRADE) **(95% CI)**	**Anticipated absolute effects** **Risk with Various controls (e.g. waitlist, standard of care, psychotherapy, yoked neurofeedback)**	**Risk difference with Neurofeedback** (95% CI)
**Clinician administered PTSD scale pre-post**	221 (7 studies)	⊕⊕⊕⊕ **HIGH**		The mean clinician administered ptsd scale pre-post in the intervention groups was **0.74 standard deviations higher** (0.11 to 1.37 higher)
**Clinician administered PTSD scale pre-FU**	103 (3 studies) 1-3 months	⊕⊕⊕⊕ **HIGH**1 due to large effect		The mean clinician administered ptsd scale pre-fu in the intervention groups was **0.8 standard deviations higher** (0.23 to 1.37 higher)
**Beck Depression Inventory pre- post**	95 (4 studies)	⊕⊕⊕⊝ **MODERATE**2 due to risk of bias		The mean beck depression inventory pre-post in the intervention groups was **0.59 standard deviations higher** (0.18 to 1.01 higher)
**PCL-5 pre-post**	166 (7 studies)	⊕⊕⊕⊕ **HIGH**		The mean pcl-5 pre-post in the intervention groupswas **0.47 standard deviations higher** (0.16 to 0.78 higher)
**PCL-5 pre-FU**	117 (5 studies)	⊕⊕⊕⊕ **HIGH**		The mean pcl-5 pre-fu in the intervention groups was **0.67 standard deviations higher** (0.27 to 1.06 higher)
*The basis for the **assumed risk** (e.g. the median control group risk across studies) is provided in footnotes. The **corresponding risk** (and its 95% confidence interval) is based on the assumed risk in the comparison group and the **relative effect** of the intervention (and its 95% CI). **CI:** Confidence interval;				
GRADE Working Group grades of evidence **High quality:** Further research is very unlikely to change our confidence in the estimate of effect. **Moderate quality:** Further research is likely to have an important impact on our confidence in the estimate of effect and may change the estimate. **Low quality:** Further research is very likely to have an important impact on our confidence in the estimate of effect and is likely to change the estimate. **Very low quality:** We are very uncertain about the estimate.				
1 Standardized mean difference using Hedges' g was 0.80 signifying a large effect szie 2 Risk of bias tended toward uncertainty				

No funnel plots to assess publication bias were generated due to the number of studies being <10, which is the minimum number recommended in order to do so (18).

## Discussion

The findings from the 17 studies included in the review suggest that NF improves PTSD symptoms no matter the instrument used and does so mainly in adults (as only three studies evaluated children/adolescents; see recommendations below). Pooled data used in meta-analyses showed mainly an effect size of ≥0.5, meaning there is likely observable clinical effect using the poolable data from the health instruments utilized. Two GRADE analyses in particular, CAPS-5 and PCL-5, pretest–posttesting, demonstrated a high quality of evidence that NF has a positive effect in treating PTSD. One GRADE analysis, CAPS-5 pre and follow-up testing, also demonstrated a high quality of evidence that NF has an effect in treating PTSD. One of the benefits in using NF is that it has been studied as an adjunct with existing therapies—with one US Food and Drug Administration (FDA) cleared technology (GrayMatters Health, 510K#K222101), which is indicated for use as an adjunctive therapy with other therapies such as psychotherapy and pharmacotherapy (42). FDA cleared is defined as the FDA allowing a device to the market through the 510(k) process based on substantial equivalence to a legally marketed predicate device. This benefit allows existing therapy(s), which a patient and clinician may be accustomed to and feel are appropriate, to be supplemented with NF.

Neurofeedback (NF) therapy trains the brain utilizing rewards to modify behavior (termed operant conditioning) through in-the-moment displays of brain activity in order to teach individuals how to self-regulate what is happening in their brain. This brain function is commonly captured *via* electroencephalogram (EEG), an accessible, low-cost technology. The major drawback of existing EEG neurofeedback methods lies in the fact that EEG signals have low spatial resolution (43) and do not capture neural activity from the deeper portions of the brain associated with processes affecting PTSD symptoms—i.e., amygdala. What has been interesting to find in the current systematic review and meta-analysis is the positive effect on outcomes “simple” EEG has despite this low spatial resolution.

Current US guidelines consider psychotherapeutic and pharmacologic therapies as the standard of care for treating PTSD (44). However, recent meta-analyses show that only 30% to 60% of patients achieve remission with psychotherapy and a significant proportion continue to have substantial residual symptoms (45). Additionally, psychotherapy can be a long process (46) and some therapies (e.g., trauma-focused CBT) require patients to reexperience their trauma (46)—leading to patient attrition (47) or to a reluctance in initiating CBT. With pharmacologic therapies for PTSD, patients may have higher dropout rates vs. psychotherapy based on unwanted side effects from the drugs (48). Evidence for the effectiveness of selective serotonin reuptake inhibitors (SSRIs) is heterogenous, independent of the duration of PTSD (49). Other pharmacologic agents including antipsychotics have limited benefits and unfavorable side-effect profiles for treating PTSD (50). This raises the importance of alternative and complementary/adjuvant therapies such as NF.

In examining heterogeneity in the CAPS-5 pre–post assessment and in pre-FU meta-analyses, removal of Fruchtman-Steinbok (16) and van der Kolk (28) reduced heterogeneity significantly. Both of these studies had effect sizes >0.80. A large effect size is defined as >0.80 (18), meaning that the findings are clearly apparent and have practical significance (51). Both of these studies also exhibited statistical significance (P < 0.05)—meaning that the intervention worked and, based on their effect size, worked well. Of interest is that the Fruchtman-Steinbok study utilized a novel fMRI-informed EEG-NF technology, which integrates simultaneous EEG and fMRI recordings (termed: Amygdala-derived EEG-fMRI-Pattern [EFP]) (7, 14). Areas of the deeper brain such as the amygdala are considered to play an important role in PTSD symptomatology (52). This technology as mentioned above has recently been cleared by the FDA for use. As it relates to van der Kolk (28) in the CAPS-5 pre-FU meta-analysis, patients treated with NF in this study (unlike the other studies in the meta-analysis) were all from the military and exhibited chronic and severe PTSD. There was a significant improvement in the CAPS-5 scores pre and FU using NF (average 40-point reduction in the CAPS-5 score). It has also been noted that military patients demonstrate smaller posttreatment improvements than civilians on various PTSD instruments in using non-NF therapies (53), and because of this, there is a need for developing new treatments, such as NF, due to this (54).

The dropout rate of those treated with NF in the systematic review and meta-analysis was 13.2%. This included some NF therapies, which required participants to relive their trauma. A recent meta-analysis on dropout rates of psychological therapies found that those with a trauma focus were significantly associated with a greater dropout rate than those without a trauma focus (18% vs. 14%) (49). Perhaps NF therapy without a trauma focus may help in this regard. Such a NF therapy for PTSD currently exists and is FDA cleared (42).

Considering that the prior systematic review (widely referenced and consisting of only four studies (1) has been used by insurance companies in making coverage determinations, the current analysis (which includes 17 studies) is needed and may assist specialty societies in establishing updated clinical guidelines around the use of NF. Additionally, these recent studies evaluate newer and more accurate deeper brain process neurofeedback (i.e., amygdala biomarkers) which affect PTSD. These newer NF studies identified in **Table 1** have positively affected PTSD symptomatology. Another positive finding is that 45% of the patients identified in the RCTs were women. The prior meta-analysis identified only 32% as female in their 123 patient meta-analysis (1).

Follow-up assessments post end of therapy for CAPS-5, PCL-5, and BDI demonstrated a prolonged/stronger effect of NF therapy compared with the completion of therapy (**Figures 5** and **7** for CAPS-5 and PCL-5). This may be due to a learned and practiced technique which patients acquire while in NF therapy. Military-related PTSD treatment has remission rates of only 40% no matter the therapy used (trauma-focused cognitive behavioral therapy, pharmacotherapy, psychotherapy) (55). The use of NF as adjunctive therapy may have the potential to improve upon these remission rates and over time.

In the majority of the RCT’s identified for this systematic review and meta-analysis, a power calculation was not undertaken. However, in six studies, it was calculated (28, 31, 33, 35–38). Four of the six studies listed calculated the sample size to identify a medium effect size (e.g., Cohen’s d ~ 0.5). The sample sizes were calculated to be 30–40 total patients in these four studies. The average size of the studies included in the meta-analysis was 36.9 ± 18. A sensitivity analysis on the sample sizes required for each study in order to arrive at a Cohen’s d of 0.5 and 0.8 is found in **Supplementary Data Sheet 5**. In order to demonstrate a large effect size (>0.8), the average study size would need to be 56 ± 8.4 patients at 80% power.

When examining active and passive controls vs. NF group differences tended to be larger in studies utilizing passive controls vs. active controls and was in line with prior findings when using NF in the treatment of major depressive disorder (MDD) (20). Passive controls can identify whether NF has a clinical benefit when used as an adjunctive therapy to standard of care (20), which was identified herein. Specific goals of neurofeedback studies are important in the choice of a control condition. If the goal is to identify clinical efficacy, a comparison with TAU/standard of care (SOC) may be appropriate (56). With sham feedback, participants are commonly provided with feedback information that is not their brain signal (and is the experimental participant’s when using yoked feedback). The benefits of a sham control include matching the experimental condition except that of gaining control of the region of interest (ROI) signal—thus, there should be equal motivation and perceived success between groups (56). Sham controls, however, might also be too conservative, as critical regions of the brain might be trained *via* functional connectivity (functional connections in the brain) (56).

An additional question, as alluded to in the introduction, is how do the protocols of the RCTs evaluated in this systematic review and meta-analysis follow the neuroscientific rationale for using NF in treating PTSD? Five of the 17 studies evaluated the use of fMRI NF with and without concomitant EEG (16, 30, 32, 34, 38). If one examined the effect of fMRI NF ± EEG on CAPS-5 reduction in those studies, which treated patients over a “standard course” of feedback (12–15 sessions), the effect size of this feedback therapy was 0.95; 95% CI (0.16 to 1.74); P = 0.02; *I* ^2^ = 80% (standardized mean difference). Additionally, a number of the studies included in this review also discussed the effect of NF using (EEG assessment) for the assessment of limbic system activity (28, 31, 33, 36, 39, 40). Thus, there is the rationale that the limbic system areas should be a focus of assessment when using NF. It has been found that the NF manipulation of specific structures of the limbic system (resulting in either increased activity or decreased activity) led to reductions in CAPS-5 scores (32, 57).

### Potential clinical effect of the above findings

A minimal clinically important difference (MCID) represents a change considered meaningful and worthwhile by a clinician in a patient’s health (58). A recent study evaluating a MCID in assessing outcomes of PTSD identified an MCID (as reported by clinicians) of between 0.758 and 0.807 (standardized mean difference or Cohen’s d) for CAPS-5, and for PCL-5 between 0.483 and 0.548 (for Cohen’s d) (58). The findings herein for both CAPS-5 (pre–post and pre-follow-up) and PCL-5 (pre-post and pre-follow-up) using Hedges’ g fall within these ranges at 0.74–0.80 and 0.47–0.67, respectively. Hedges’ g was used in the current analysis due to small sample sizes found in the studies—in order to reduce positive bias (59).

### Limitations of analysis

Only English language articles were identified. This is not to say that foreign language RCTs do not exist. Cost effectiveness was only evaluated in only one study with the quality of life instrument assessed over 1 year only. Therefore, while the cost effectiveness may have been acceptable at $15,600 (60), additional data on quality of life beyond 1 year and costs beyond 1 year would have been more informative.

### Strengths of analysis

Every instrument utilized identified NF as the statistically significant more clinically efficacious therapy vs. control. As well, PTSD symptomatology improved after completion of NF therapy as measured by CAPS-5 and PCL-5. This analysis also includes female patients, which comprised 45% of those studied. This analysis also appears to be unique in that it evaluates depressive symptom improvements using standardized effect sizes (for BDI outcome). In a prior systematic review on the use of NF for major depressive disorder (MDD) (20), most studies showed symptom improvement superior to controls with the caveat that most articles did not comply with stringent study quality and reporting practices. While the intention of the current analysis was to focus on PTSD symptomatology, MDD is a common comorbidity with PTSD.

Since this analysis was exclusively based on RCTs that tested the efficacy of NF in treating PTSD, best practices as per (CRED-nf) were complied with/observed in relation to having a control group, defining a feedback modality as part of the methodology and in the reporting of outcomes. The addressing of these criteria differed meaningfully from other best practice analyses (20, 61, 62)—mainly due to the fact that these other best practice analyses included non-RCTs.

### Suggested future directions for research

A number of the NF studies evaluated used NF in conjunction with other evidence-based clinical practice recommendations—i.e., psychotherapy and SSRIs (63). This may have been due to the chronicity of the PTSD condition and patients being refractory to the evidence-based practice recommendations. Several issues were identified in this systematic review and meta-analysis for future research and as in previous systematic reviews in other areas (20, 61, 62). These issues along with suggested recommendations for future research are listed in **Table 6** below.

**Table 6:** Summary issues and recommendations for future research.

Identified issue	Suggested recommendations
NF has mainly been evaluated as an adjunct therapy to psychotherapy and pharmacotherapy (evidence-based guideline therapies)_	RCT examining NF as a first-line therapy compared with either guideline therapies or NF compared with NF + adjunct.
Lack of a power calculation for sample size	Ensure investigational device exemption (IDE)-approved NF trials include a power calculation.
Lack of blinding of patient and clinician	Ensure at the very least that the clinical assessor is blinded to participant treatment allocation—especially with TAU
Lack of collection of brain activity used for feedback to experimental participants	Collect brain activity and provide as part of the reported findings.
Mild to moderate PTSD evaluated in the majority of trials.	Inclusion of severe PTSD patients in trials.
Durability of clinical efficacy posttreatment	Follow-up with patients over a longer term than 3 months.
Lack of children/adolescents (3 of 17 trials)	Include more studies examining the effect of NF on PTSD in children adolescents.
Justification and reporting of reinforcement schedule	Include justification and reinforcement schedule as part of the methods section.
Reporting how patients responded within and between sessions	Include in the results section/appendices how patients responded within and between session.

Lastly, as part of moving the neurofeedback field/technology forward, preregistering clinical trial information on various national databases—e.g., ClinicalTrials.gov (as a national clinical trial or NCT)—enhances public trust by creating a transparent public record of clinical trials and information about their results and it permits the scientific community to build on information made available. Unfortunately, only eight of the 15 identified studies were listed on these types of databases. A listing of these studies appears as **Supplementary Data Sheet 3**.

## Conclusions

The level of evidence in adults (14 studies) suggests that NF helps adult PTSD, but there is a lack of data to drive conclusions in children/adolescents (only three studies). Neurofeedback as an adjunctive therapy to psychotherapy and/or pharmacotherapy has demonstrated clinically meaningful changes (based on Hedges’ g) in the eyes of patients and/or experienced providers (58) in lowering PTSD symptomatology in this systematic review and has done so in follow-up after therapy has ended—demonstrating durability of treatment. Neurofeedback should be more widely available—especially when used in conjunction with evidence-based therapies. Updating insurance coverage policies to include NF as a covered therapy for PTSD should be revisited based on these findings. Furthermore, updating clinical guidelines for the treatment of PTSD should also be considered with the option of using NF adjunctively. The introduction of fMRI NF and fMRI-informed EEG NF add to the body of evidence that NF is clinically efficacious in treating PTSD.

A protocol was not prepared for this analysis.

## Data availability statement

The original contributions presented in the study are included in the article/**Supplementary Material**. Further inquiries can be directed to the corresponding author.

## Author contributions

JV: Conceptualization, Data curation, Formal analysis, Investigation, Methodology, Writing – original draft, Writing – review & editing. MM: Formal analysis, Methodology, Writing – review & editing. AT: Writing – review & editing.
