# Supplementary material for: Systematic review and meta-analysis of neurofeedback and its effect on posttraumatic stress disorder
Source: Front Psychiatry. 2024 Mar 21;15:1323485. doi: 10.3389/fpsyt.2024.1323485 (PMC10993781; doi:10.3389/fpsyt.2024.1323485)
Supplement: Supplementary file 1 [file DataSheet_1.pdf]

Neurofeedback for PTSD

Characteristics of studies

Characteristics of included studies

Antle 2018

**Methods**	Single center randomized controlled trial at a school in Pokhara, Nepal. Unclear as to the dates the trial took place
**Participants**	Twenty one girls living in poverty (aged 5-11). All suffered trauma resulting form violence in home, substance abuse in the home, neglect, and/or parental death.
**Interventions**	Intervention group (n=9) Mind-Full consisting of 3 simple games - based on familiar, everyday activities and actions which when learned, elicit behaviors resulting in desired brain states related to relaxation or attention. Use of a EEF headset monitors brainwave activiity and uses pre-processed data outputs on a tablet for either relaxation or attention - this provides visual feedback to children on the relaxation or attentive state and; provides guidance and motivation to change their brain states. This included 6 weeks of 4 sessions per week. Waitlist (n=12)
**Outcomes**	Measures of anxiety and attention in observable behavior at pre-test, post-test and follow-up (2 months post test) using a survey instrument provided administered to the children by counselors and teachers. Determination of whether children were able to transfer their self-regulatory behaviors learned through Mind-Full into the classroom and playground.
**Notes**	

Risk of bias table

**Bias**	**Authors' judgement**	**Support for judgement**
Random sequence generation (selection bias)	Low risk	Girls randomly assigned to intervention or waitlist
Allocation concealment (selection bias)	Unclear risk	Unclear as to when children were allocated to each group and when study begaan
Blinding of participants and personnel (performance bias)	High risk	Teachers and counselors knew to which group the children were assigned.
Blinding of outcome assessment (detection bias)	High risk	Clinical assessor knew to which group the children were assigned.
Incomplete outcome data (attrition bias)	Low risk	All 21 children completed the study.

Selective reporting (reporting bias)	Low risk	All outcomes as outlined in the methods section were reported on.
Other bias	Low risk	No other biases noted.

Bell 2019

**Methods**	Single center randomized trial taking place in Denver/Boulder, CO, USA. Unclear as to the dates the trial took place.
**Participants**	Adults between the ages of 18-80 who self reported an experience with a traumatic event with a PTSD Checklist (PCL-5) >20. HRV biofeedback (HRVB) (N=11): average age 43.7+/-8.8 years; LORETA Z-Score Neurofeedback [N=12]: average age 44.6+/-13.1 years. Of the 23 patients that completed the study 78% were taking psychiatric medication and 83% were receiving psychotherapeutic support. No mention of female/male breakout.<br>Exclusion criteria included: moderate to severe brain injury,current diagnosis of a personality disorder, active psychosis, active suicidal ideation, pregnancy.
**Interventions**	HRV biofeedback (HRVB) (N=11) vs. LORETA Z-Score Neurofeedback [N=12] (utilizes EEG cap with 3-D source imaging to determine the specific source of an electric dipole - allows for targeted real-time training of individual brain regions, deeper within the cortex). All participants attended 15 sessions of training for each arm of the trial at a rate of 2 sessions per week. Each session included 20 minutes of direct training, divided into 4 rounds of 5 minutes.
**Outcomes**	Psychosocial assessments (self reported): PTSD checklist for DSM-V (PCL-5) and the Beck Anxiety Inventory (BAI). Assessments took place at baseline and at the end of therapy.
**Notes**	Authors have no conflicts of interest or financial gains to report. The study was partially funded by mini-grants from the Foundation for Neurofeedback and Neuromodulation Research (FNNR) and the Foundation for Education and Research in Biofeedback and Related Sciences (FERB).

Risk of bias table

Bias	Authors' judgement	Support for judgement
Random sequence generation (selection bias)	Low risk	*Twenty four eligible adults enrolled on a first come, first serve basis and were alternately assigned between each group according to the order in which they returned the prescreening material.*
Allocation concealment (selection bias)	Low risk	Patients did not know to which group they would have been assigned as; assignment was dependent upon the order in which they returned their prescreening materials.

Blinding of participants and personnel (performance bias)	High risk	Clinicians knew which group patients were assigned.
Blinding of outcome assessment (detection bias)	Unclear risk	Unclear if those assessing the self reported results were aware of which group patients were assigned to.
Incomplete outcome data (attrition bias)	Low risk	Of 24 patients entered into the trial only one patient in the HRVB group withdrew prior to completion of the study. Attrition rate of 4.2%.
Selective reporting (reporting bias)	Low risk	All outcomes identified in the methods section were reported on in the results section.
Other bias	Low risk	*Authors have no conflicts of interest or financial gains to report.*

Fine 2023

**Methods**	Single center randomized controlled trial taking place in Tel Aviv, Israel.
**Participants**	Fifty five female chronic (29 years since trauma) childhood sexual abuse PTSD patients of average age of 36-37 years.
**Interventions**	Amygdala EEG NF [Amyg-EFP-NF] (N=40) plus psychotherapy vs. psychotherapy (N=15). Amy-EFP-NF included ten sessions adminstered twice a week for 2 weeks and then weekly (total of 10 weeks). Psychotherapy sessions occurred weekly.
**Outcomes**	Primary outcomes based on Clinician administered PTSD scale (CAPS-5) assessed at completion of therapy, 1, 3, and 6 months later. The secondary outcome included the post-traumatic checklist for DSM-5; PCL-5). The dissociation measure (dissociation experieces scale. DES-II) was an exploratory endpoint.
**Notes**	Listed on Clinical trials.gov NCT03416764; Sent email to corresponding author on October 4, 2023 regarding additional data on outcomes for 1, 3, 6 months.

Risk of bias table

**Bias**	**Authors' judgement**	**Support for judgement**
Random sequence generation (selection bias)	Low risk	Randomized controlled trial.
Allocation concealment (selection bias)	Low risk	*Randomization took place immediately before the start of treatment* per email from lead author on October 5, 2023.
Blinding of participants and personnel (performance bias)	High risk	Both patients and clinicians knew to which treatment group patients were assigned.
Blinding of outcome assessment (detection bias)	Low risk	*PTSD symptoms were blindly assessed pre-and post NF training period.*

Incomplete outcome data (attrition bias)	Low risk	Of the 40 in the Amyg-EFP-NF group, 39 were assessed post treatment (1 dropout). Of the 15 in the psychotherapy group 14 were assessed post treatment (1 dropout).
Selective reporting (reporting bias)	Low risk	All outcomes listed in the methods section were assesed in the results section.
Other bias	Low risk	None noted

Fruchtman-Steinbok(2) 2021 Neu NF

**Methods**	Single center randomized controlled trial taking place in Tel Aviv, Israel. Unclear as to dates study took place. However from information provided on ClinicalTrials.gov; NCT02544971, study started on April 2016 and ended June 2019.
**Participants**	Trauma-NF: average age 40.25+/-21.96; 58.33% percent female; Neutral-NF: average age 37.66+/-10.7; 40% female; No-NF: average age 32+/-8.7; 38.46% female. All participants who were currently in psychotherapy and/or were receiving pharmacological treatments were included in the study.<br>Exclusion criteria included: pregnancy, major medical or neurological disorder, psychosis, schizophrenia, and suicidal ideation.
**Interventions**	Neutral neurofeedback (N=13) - neurofeedback guided by an amygdala-fMRI informed EEG model of amygdala activity termed electrical-finger-print (EFP); Trauma neurofeedback (N=12) - neurofeedback guided by an amygdala-fMRI informed EEG model of amygdala activity termed electrical-finger-print (EFP) used in conjunction with a trauma script recording. Successful downregulation of the amygdala was reflected by the reduced sound volume of the trauma recording. In both Neutral-NF and Trauma-NF during each session of 5 repetitions of 3 consecutive conditions: passive watch/baseline a busy emergency waiting room, active regulate (3 minutes) in which patients were instructed to down-regulate feedback stimuli by practicing self-generated mental images; and a debriefing with graphic feedback on signal modulation. It uses a busy and noisy waiting-room scene where the avatars tend to huddle at the reception desk and yell. The patient goal is to get the avatars to sit down, to keep them seated and calm, and to lower their voices by identifying the personal mental strategy that works for the patient. This is determined by the amygdalaEFP signal power (lower power people sit; higher power results in unrest in waiting room). Neurofeedback included 15 sessions over 13 weeks (twice weekly for 2 weeks and then once per week); No neurofeedback (N=13) - current therapy in place (e.g. psychotherapy and/or pharmacotherapy).
**Outcomes**	Primary outcomes included: CAPS-5 and PTSD checklist (PCL) for the pre and post intervention time points (before and immediately after the neurofeedback training period as well as via self report at 3 and 6 month follow-up). Secondary endpoints included: State-Trait Anxiety Inventory (STAI); Beck Depression Inventory-II (BDI-II) Toronto Alexithymia Scale (TAS-20), and Emotion Regulation Questionnaire (ERQ). Again all were measured before and immediately after the

	neurofeedback training period as well as via self report at 3 and 6 month follow-up.
**Notes**	Two authors invented related patents. All other authors declared no financial conflict of interest.

Risk of bias table

Bias	Authors' judgement	Support for judgement
Random sequence generation (selection bias)	Unclear risk	Adults meeting PTSD criteria were randomized between Trauma-NF, Neutral-NF and No-NF. Unclear however, what randomization sequence was utilized.
Allocation concealment (selection bias)	Unclear risk	Unclear as to whether patients knew beforehand to which group they would be allocated to.
Blinding of participants and personnel (performance bias)	High risk	Patients and personnel administering therapies were not blinded to treatment arms.
Blinding of outcome assessment (detection bias)	Low risk	PTSD symptoms were blindly assessed before and immediately after the neurofeedback training period as well as via self report at 3 and 6 month follow-up.
Incomplete outcome data (attrition bias)	High risk	Neutral-NF (N=19 randomized); 5 dropouts; Trauma-NF (N=20 randomized); 7 dropouts; No-NF (N=20 randomized) 7 dropouts. Attrition rate of 32.2%.
Selective reporting (reporting bias)	Low risk	All outcomes as identified in methods section were reported on in results section.
Other bias	Low risk	Two authors invented related patents. All other authors declared no financial conflict of interest.

Fruchtman-Steinbok 2021 TR-NF

**Methods**	Single center randomized controlled trial taking place in Tel Aviv, Israel. Unclear as to dates study took place. However from information provided on ClinicalTrials.gov; NCT02544971, study started on April 2016 and ended June 2019.
**Participants**	Trauma-NF: average age 40.25+/-21.96; 58.33% percent female; Neutral-NF: average age 37.66+/-10.7; 40% female; No-NF: average age 32+/-8.7; 38.46% female. All participants who were currently in psychotherapy and/or were receiving pharmacological treatments were included in the study.<br>Exclusion criteria included: pregnancy, major medical or neurological disorder, psychosis, schizophrenia, and suicidal ideation.
**Interventions**	Neutral neurofeedback (N=14) - neurofeedback guided by an amygdala-fMRI informed EEG model of amygdala activity termed electrical-finger-print (EFP); Trauma neurofeedback (N=13) - neurofeedback guided by an amygdala-fMRI

	informed EEG model of amygdala activity termed electrical-finger-print (EFP) used in conjunction with a trauma script recording. Successful downregulation of the amygdala was reflected by the reduced sound volume of the trauma recording. In both Neutral-NF and Trauma-NF during each session of 5 repetitions of 3 consecutive conditions: passive watch/baseline a busy emergency waiting room, active regulate (3 minutes) in which patients were instructed to down-regulate feedback stimuli by practicing self-generated mental images; and a debriefing with graphic feedback on signal modulation. It uses a busy and noisy waiting-room scene where the avatars tend to huddle at the reception desk and yell. The patient goal is to get the avatars to sit down, to keep them seated and calm, and to lower their voices by identifying the personal mental strategy that works for the patient. This is determined by the amygdalaEFP signal power (lower power people sit; higher power results in unrest in waiting room). Neurofeedback included 15 sessions over 13 weeks (twice weekly for 2 weeks and then once per week); No neurofeedback (N=13) - current therapy in place (e.g. psychotherapy and/or pharmacotherapy).
**Outcomes**	Primary outcomes included: CAPS-5 and PTSD checklist (PCL) for the pre and post intervention time points (before and immediately after the neurofeedback training period as well as via self report at 3 and 6 month follow-up). Secondary endpoints included: State-Trait Anxiety Inventory (STAI); Beck Depression Inventory-II (BDI-II) Toronto Alexithymia Scale (TAS-20), and Emotion Regulation Questionnaire (ERQ). Again all were measured before and immediately after the neurofeedback training period as well as via self report at 3 and 6 month follow-up.
**Notes**	Two authors invented related patents. All other authors declared no financial conflict of interest.

Risk of bias table

Bias	Authors' judgement	Support for judgement
Random sequence generation (selection bias)	Unclear risk	Adults meeting PTSD criteria were randomized between Trauma-NF, Neutral-NF and No-NF. Unclear however, what randomization sequence was utilized.
Allocation concealment (selection bias)	Unclear risk	Unclear as to whether patients knew beforehand to which group they would be allocated to.
Blinding of participants and personnel (performance bias)	High risk	Patients and personnel administering therapies were not blinded to treatment arms.
Blinding of outcome assessment (detection bias)	Low risk	PTSD symptoms were blindly assessed before and immediately after the neurofeedback training period as well as via self report at 3 and 6 month follow-up.
Incomplete outcome data (attrition bias)	High risk	Neutral-NF (N=19 randomized); 5 dropouts; Trauma-NF (N=20 randomized); 7 dropouts; No-NF (N=20 randomized) 7 dropouts. Attrition rate of 32.2%.

Selective reporting (reporting bias)	Low risk	All outcomes as identified in methods section were reported on in results section.
Other bias	Low risk	Two authors invented related patents. All other authors declared no financial conflict of interest.

Kelson 2013

**Methods**	Randomized controlled trial of veterans with PTSD that took place at The Salvation Amry Bell Center, CA. Unclear as to dates the trial took place.
**Participants**	Homeless and near homeless male veterans (age range 30-62 years) living at The Salvation Army Bell Center. Nine of the 10 participants has histories of alcoholism and drug abuse.
**Interventions**	EEG Biofeedback of 20 sessions (30 minutes per session) over a 4 week period vs. no treatment (control).
**Outcomes**	Mean scores from the Likert rating scale (1-5) of the 23 PTSD Symptoms Questionnaire. These scores were evaluated at baseline and at the end of every week of the study.
**Notes**	

Risk of bias table

**Bias**	**Authors' judgement**	**Support for judgement**
Random sequence generation (selection bias)	Unclear risk	*Random selection of veterans in the treatment group and no treatment group and gave the two lists to the researcher.* Unclear as to randomization scheme.
Allocation concealment (selection bias)	High risk	Participants knew beforehand to which group they had been assigned.
Blinding of participants and personnel (performance bias)	High risk	Participants and personnel administering the therapy knew to which group each belong.
Blinding of outcome assessment (detection bias)	High risk	Statisitician and study coordinator knew to which group each belonged.
Incomplete outcome data (attrition bias)	Low risk	14 were randomized and 10 completed the study. 29% attrition rate.
Selective reporting (reporting bias)	Low risk	All outcomes as identified in the methods section were reported on in the results section.
Other bias	Low risk	

Leem 2021

**Methods**	Randomized, wait-list controlled, single center, assessor-blinded at Wonkwang University Sanbon Hospital, South Korea, undertaken from May 2019 to September 2020.
**Participants**	All patients diagnosed with PTSD. Experimental group (N=10): Average age of 44.4+/-13.6 years; 9 female/1 male. Control group (N=9): Average age of 43.6+/-19.1 years of age; 8 female/1 male.<br>Exclusion criteria included: severe mental disease, suicidality, pregnancy, when it is necessary to administer continuously a substance that is judged to have an effect on the induction of PTSD symptoms, psychotherapy.
**Interventions**	Experimental group (N=10): received 16 neurofeedback self regulated training (NSFT) based on quantitative EEG analysis over a period of 12 weeks. Neurofeedback utilized alpha-theta brainwave neurofeedback (goal is to strengthen alpha-theta waves and suppress beta waves). Control group waited for 12 weeks while experimental group was being treated. Both experimental and control group were allowed to continue on any drugs on non-pharmacological treatments (other than psychotherapy).
**Outcomes**	Primary outcome: PTSD Checklist for DSM-5 (PCL-5) [Korean version] - measured at baseline, week 4, week 8 (end of therapy) and week 12 (one month after therapy ends) Secondary outcome measures include and were measured at baseline, week 4, week 8 (end of therapy) and week 12 (one month after therapy ends): Impact of the event scale revised [Korean version]; Clinical Global Impression Improvement Scale (GCI-I); Beck Anxiety Inventory (BAI); Beck Depression Inventory (BDI); Insomnia Severity Index (ISI); Hwa-Byung Scale (HBS); Core seven emotions inventory short form (CSEI-5). Mentalizing the rooms of mind (MRM) were evaluated before and after each NSFT visit. QEEG analysis was assessed at every visit. Adverse events were recorded and categorized at mild, moderate, or severe.<br>Quality of life assessments included: Short form health survey-36 (SF-36) and the EuroQol-5 Dimension (EQ-5D-5L) were assessed at baseline at the end of therapy (week 8) and one month after therapy ended (week 12).<br>Cost outcomes included direct medical and non-medical costs (travel and indirect [loss of productivity] were also evaluated at baseline at the end of therapy (week 8) and one month after therapy ended (week 12).
**Notes**	*Authors declared no conflicts of interest.*

Risk of bias table

**Bias**	**Authors' judgement**	**Support for judgement**
Random sequence generation (selection bias)	Low risk	*Randomization table developed by an independent statistician using block randomization methods in Microsoft Excel.*

Allocation concealment (selection bias)	Low risk	*Independent clinical research coordinator allocated the participants according to the randomization table to maintain allocation concealment.*
Blinding of participants and personnel (performance bias)	High risk	*Due to the wait list control group design, the participants and neurofeedback practitioners were not blinded.*
Blinding of outcome assessment (detection bias)	Low risk	*Clinical assessor and statistician were blinded.*
Incomplete outcome data (attrition bias)	Unclear risk	Of the 22 patients entered into the trial, one was lost to follow-up in the NFST group and there were 2 withdrawals in the wait list group. The attrition rate was 13.6%. (It has been published previously that attrition under 5% is not likely to introduce bias, while attrition rates above 20% raise concerns about the study validity;Babic, A., Tokalic, R., Amílcar Silva Cunha, J. *et al.* Assessments of attrition bias in Cochrane systematic reviews are highly inconsistent and thus hindering trial comparability. *BMC Med Res Methodol* **19,** 76 (2019). https://doi.org/10.1186/s12874-019-0717-9)
Selective reporting (reporting bias)	Low risk	All outcomes as listed in the methods section were reported on in the results section.
Other bias	Low risk	*The authors declare no conflict of interest.*

Misaki 2018

**Methods**	Prospective randomized controlled study conducted at the Laureate Institute for Brain Research, Tulsa, Oklahoma, United States. Unclear as to the dates of the trial.
**Participants**	Male combat veterans (18 to 55 years old) who met the criteria for PTSD. Exclusion criteria included serious suicidal ideation, psychosis, major medical or neurological disorders, MRI exclusions and exposure to psychotropic medications, current or past history of schizophrenia, schizoaffective disorder, biopolar disorder, or dementia, moderate or severe traumatic brain injury (TBI)
**Interventions**	Left amygdala rtfMRI-neurofeedback positive emotion training conducted at the 3rd, 4th, and 5th visits out of a total of 7 visits. Days between the 7 visits were generally 11-14 days. Participants instructed to recall a positive autobiographical memory to increase the neurofeedback signal (N=15). Control group who received sham neurofeedback from a region not involved in emotion processing (N=8).
**Outcomes**	Clinician administered PTSD scale (CAPS) - administered at baseline and at the last of 7 total visits. Montgomery-Åsberg Depression Scale (MADRS) including a structural MRI scan at the 2nd and 6th visits.

**Notes**	*The authors report no conflicts of interest.* Note this study is a duplicate of Zotev et al. Real-time fMRI neurofeedback training of the amygdala activity with simultaneous EEG in veterans with combat-related PTSD. NeuroImage Clinical 2018.19:106-121. As such data from the Zotev study was combined with Misaki 2018. As well this study was followed up on and published in 2021: Misaki M et al. Hippocampal volume recovery with real-time functional MRI amygdala neurofeedback emotional training for posttraumatic stress disorder. Jrl Affect Dis. 2021;283:229-235. Information from this study was added to Misaki 2018. These duplicate publications were confirmed in an email from Masaya Misaki, PhD on July 31, 2023. In the current systematic review and meta-analysis, it was decided per discussion with the co-authors, to use the Zotev 2018 patient and outcome data on fMRI-nf as it more closely mirrored other therapies in the systematic review and meta-analysis. Per Cochrane guidance on referencing, this also necessitated using the first publication (Misaki 2018) as the reference study.

Risk of bias table

Bias	Authors' judgement	Support for judgement
Random sequence generation (selection bias)	Unclear risk	*Participants were randomly assigned in a 2:1 ratio to either the experimental group who received active neurofeedback from the left amygdala or the control group who received sham neurofeedback from a region not involved in emotion processing.* Unclear however as to how randomization sequence generation occurred.
Allocation concealment (selection bias)	Unclear risk	Unclear as to whether participants knew which group they would be assigned to during randomization.
Blinding of participants and personnel (performance bias)	Unclear risk	*Participants were blind to group assignment.* Unclear as to whether clinicians administering the interventions were blinded.
Blinding of outcome assessment (detection bias)	Unclear risk	Unclear as to whether those assessing the instruments and performing and assessing the MRIs were aware of assignment.
Incomplete outcome data (attrition bias)	Unclear risk	Of 25 assigned to the experimental arm, 4 did not complete the 7 sessions. Of 11 assigned to the control arm, 2 did not complete the 7 sessions - 17% attrition rate.
Selective reporting (reporting bias)	Low risk	All outcomes as identified in the methods section were reported on in the results section.
Other bias	Unclear risk	*The authors report no conflicts of interest.*

Nicholson 2020

**Methods**	Double blind randomized controlled trial - single center, trial took place from 2014 to 2018 in Ontario, Canada.
**Participants**	All patients diagnosed with PTSD. Experimental group (N=20) consisted of 13 females/7 males with an average age of 39.2+/-12.1 years. The control group (N=18) consisted of 14 females/4 males with an average age of 46.3+/-12.4 years. Twelve patients in each group were on psychotropic medications. Exclusion criteria included: alcohol dependency or substance use disorder, past or current biofeedback treatment, acute suicidality within the past 3 months, self injurious behavior, involvement in a violent relationship, unstable living condition.
**Interventions**	All patients underwent and initial fMRI scan. Experimental group: Participants then began a 20 week (a alpha rhythm neurofeedback session each week) with pre- and post-alpha rhythm neurofeedback resting state EEG recordings. Control group: Yoked neurofeedback signal corresponded to a replayed feedback signal from a successful participant in the experimental group in order to ensure motivational states in control group. Participants in each group did not receive explicit strategies on how to down-regulate the alpha signal during the trial and were to told to explore individual strategies. Particpants completed the EEG-neurofeedback via selecting continuous visual feedback in the form of either: photo that had been divided into a grid, with individual grid pieces appearing as alpha amplitude was suppressed; or a cartoon character that moved across the screen as alpha amplitude was suppressed. Participants also received auditory feedback in the form of a series of single beeps, which occurred when they were suppressing the amplitude and corresponded to their visual feedback.
**Outcomes**	Primary outcome measure change in PTSD score as evaluated by CAPS-5. Baseline, post-treatment and 3 months post treatment CAPS were assessed.
**Notes**	Question on conflicts of interest send to author on 6/28/23 via email. Note: Lanius R et al. Regulating posttraumatic stress disorder symptoms with neurofeedback: Regaining control of the mind. Neuroreg. 2020;7(4):161 is a duplicate of Nicholson. As well Shaw SB, Nicholson AA, Ros T, et al. Increased top-down control of emotions during symptom provocation working memory tasks following a RCT of alpha-down neurofeedback in PTSD. Neuroimage Clin. 2023;37:103313 is a duplicate of Nicholson 2020. Further, Nicholson AA, Densmore M, Frewen PA, Neufeld RWJ, Théberge J, Jetly R, et al. (2023) Homeostatic normalization of Alpha brain rhythms with the default-mode network and reduced symptoms in post-traumatic stress disorder following a randomized controlled trial of electroencephalogram neurofeedback. Brain Comm. https://doi.org/10.1093/braincomms/fcad068 is a duplicate of Nicholson 2020. As such, the studies were combined to extract maximal information. Reply email from R. Lanius on July 14, 2023 and on September 6, 2023. Reply emails from A. Nicholson on December 16, 2023

Risk of bias table

Bias	Authors' judgement	Support for judgement
Random sequence generation (selection bias)	Low risk	Double blind randomized controlled trial to either the EEG-neurofeedback or sham-control EEG-neurofeedback group. Email to author on 6/28/23 to determine randomization scheme. Reply email from R. Lanius on July 14, 2023 - coin flip.
Allocation concealment (selection bias)	Low risk	Sent email to authors on 6/28/23 seeking info on allocation concealment.Reply email from R. Lanius on July 14, 2023 - patients were unaware.
Blinding of participants and personnel (performance bias)	Low risk	Double blinded trial (participants and clinicians administered neurofeedback.
Blinding of outcome assessment (detection bias)	Low risk	Sent email to authors on 6/28/23 seeking if outcome assessor was blinded to treatment arms. Reply email from R. Lanius on July 14, 2023 - clinical assessors were blinded.
Incomplete outcome data (attrition bias)	Low risk	A total of 4 patients out of the 40 PTSD enrolled patients were excluded due to incomplete fMRI resting scans. Attrition rate of 10%.
Selective reporting (reporting bias)	Low risk	All outcomes as identified in the methods section were reported on in the results section.
Other bias	Low risk	Question on conflicts of interest to author on 6/28/23.Reply email from R. Lanius on July 14, 2023 - no conflicts of interest.

Noohi 2017

**Methods**	Single center randomized controlled trial of patients diagnosed with PTSD. Clinical trial took place in Iran. Unclear if this was a single or multi-center trail. Unclear as to dates of when trial was performed.
**Participants**	Patients with diagnosed PTSD. Participants were between 25-60 years of age.
**Interventions**	Experimental group (N=15) treated with neurofeedback with alpha/theta method for 25 sessions each lasting 30-40 minutes for 4 times per week. Patients were provided with audio (river and ocean waves) and then asked to recall positive memories. Control group (N=15) - unclear as to what they received for treatment. It was assumed no treatment was provided.
**Outcomes**	Impact of event scale-revised (IES-R) - measures PTSD symptoms in response to a specified trauma. Beck depression inventory-II; Wisconsin card sorting test (WCST) - measures executive function; Tower of London (ToL) - measures executive function. All of these outcomes were measured at baseline, end of treatment (6 weeks) and 45 days after final treatment session.
**Notes**	No competing interests declared by authors.

Risk of bias table

Bias	Authors' judgement	Support for judgement
Random sequence generation (selection bias)	Unclear risk	Randomized controlled trial. Unclear as to randomization scheme used.
Allocation concealment (selection bias)	Unclear risk	Unclear as to whether participants knew to which group they were assigned.
Blinding of participants and personnel (performance bias)	Unclear risk	No mention as to whether participants or personnel performing neurofeedback were blinded to treatment arms.
Blinding of outcome assessment (detection bias)	Unclear risk	No mention as to who administered outcome tests.
Incomplete outcome data (attrition bias)	Low risk	All patients entered into trial completed trial.
Selective reporting (reporting bias)	Low risk	All outcomes identified in methods section were reported on in results section.
Other bias	Low risk	No competing interests declared by authors.

Onton 2016

**Methods**	Randomized trial that took place at the United States Naval Medical Center, San Diego, CA from May 2012 to August 2016.
**Participants**	Patients appeared to have multiple behavioral health conditions including: PTSD, anxiety, and sleep disorders. EEG biofeedback group: average age:30.4+/-5.2 years; 22 males/2 females; Sham-biofeedback group: average age: 29.4+/-5.6 years; 21 males/3 females.
**Interventions**	EEG biofeedback over a 4 week period vs. Sham/yoked EEG biofeedback (patient receives feedback according to someone else's brain rhythms collected during a different session) over the same period. Unclear as to the number of sessions performed.
**Outcomes**	Improved symptom rating on anxiety and irritability. The scale for each rating was a 0-10, with a 0 meaning not at all and 10 being extremely anxious/irritable.
**Notes**	Sent email to author of NCT01591408 on 6/30/23 seeking additional information on trial.

Risk of bias table

Bias	Authors' judgement	Support for judgement
Random sequence generation (selection bias)	Unclear risk	States patients were randomized but unclear as to randomization scheme.
Allocation concealment (selection bias)	Low risk	Both EEG and Sham-EEG group were similar in delivery, assumes patient was unaware of what group they were assigned to.
Blinding of participants and personnel (performance bias)	Unclear risk	Both EEG and Sham-EEG group were similar in delivery, assumes patient was unaware. However, unclear if clinician administering the EEG was aware of the real or sham group.
Blinding of outcome assessment (detection bias)	Unclear risk	Unclear as to who evaluated the data and if they were aware of treatment allocation assignment.
Incomplete outcome data (attrition bias)	High risk	68 patients entered the trial. 48 completed the intervention. 29% attrition rate.
Selective reporting (reporting bias)	Low risk	Improved symptom rating identified was reported on in the results.
Other bias	Low risk	None noted

Peniston 1991

**Methods**	Single center randomized trial taking place at Ft. Lyon VA Medical Center, Ft. Lyon Colorado, USA. Unclear as to dates the trial took place.
**Participants**	Twenty 29 male Vietman veterans with a 12-15 year history of chronic combat related PtSD and with frequent recurring combat-related nightmares/flashbacks that were anxiety provoking events. Experimental group had a mean age of: 36.1+/-2.6 years. Control group had a mean age of 37.3+/-2.8 years.<br>Exclusion criteria: evidence of psychotic symptoms; organic dysfunctions
**Interventions**	Experimental group: EEG alpha-theta brainwave neurofeedback training (BWT) (N=15). Thirty 30-minutes sessions over a period of 28 days were provided (BWT patient seen 5 days per week). Patients were followed up monthly for a period of 30 months after completion of BTW. During BTW subjects were instructed to close their eyes and construct visualized scenes of their nightmares and flashbacks. They were then instructed to visualize imageries of increased alpha rhythm amplitude and scenes of the normalization of their personalities, keeping the mind quiet and alert and the body calm. Control group: traditional medical control group given psychotropic medications and combined individual and group psychotherapy.
**Outcomes**	Primary outcome: Minnesota Multiphasic Personality Inventory (MMPI) used to assess personality changes. MMPI was administered at baseline and end of therapy. Other outcomes measured: medication consumption measured weekly over BWT therapy and as well with control group.

**Notes**	

Risk of bias table

Bias	Authors' judgement	Support for judgement
Random sequence generation (selection bias)	Unclear risk	*Subjects randomly assigned to either BTW or traditional medical control.* Unclear however as to randomization scheme.
Allocation concealment (selection bias)	Unclear risk	No mention as to whether patients knew ahead of time to which treatment group they would be allocated to.
Blinding of participants and personnel (performance bias)	High risk	Physicians treating patients at the VA hospital were aware of the treatment groups.
Blinding of outcome assessment (detection bias)	Unclear risk	Unclear as to whether clinicians/statisticians evaluating the results were aware of treatment arms.
Incomplete outcome data (attrition bias)	Low risk	All patients who entered trial completed it.
Selective reporting (reporting bias)	Low risk	All outcomes identified in methods section were reported on in results section.
Other bias	Low risk	None noted.

Rogel 2020

**Methods**	Single center randomized controlled trial conducted in Boston, MA, USA (Trauma Center, Justice Resource Institute), from February 1, 2014 to Janaury 31, 2017.
**Participants**	Children aged 6-13 years of age who met the following criteria: Two or more interpersonal traumatic experiences; weekly individual therapy with the same therapist for at least 3 months prior to study; no medication or psychosocial treatment changes in the past 3 months; diagnosis of PTSD. Majority of children were adopted (N=28).<br>Exclusion criteria: history of epilepsy, seizure or head injury; currently on benzodiazepines.
**Interventions**	Experimental: Neurofeedback group (N=20) received neurofeedback twice a week for 24 sessions over course of 12 weeks; average age of 9.65+/-1.7 years; 15 males/5 females. During each session, brain electrical activity was recorded while participants watched a computer game that reflected the status of their EEG activity. If the power of the recorded brain signals at the specific frequencies (bands) were met, participants were rewarded with audio and visual signals.<br>Control: wait-list group continued to receive treatment as usual; average age of 9.6+/-2.1 years; 9 males/8 females.

**Outcomes**	Child Behavioral Check List (CBCL); Behavior Rating Inventory of Executive Function (BRIEF); Trauma Symptom Checklist for Young Children (TSCYC); Children's Alexithymia Measure (CAM); Child Behavior Checklist (CBCL); PTSD Reaction Index (PSTD-RI); Child Dissociative Checklist, Kiddie Schedule for Affective Disorders (K-SADS); NIH Toolbox cognitive battery, Caregiver neurofeedback symptom checklist; Child neurofeedback symptom checklist, Children's Depression Inventory 2. All were to be administered before treatment, immediately after treatment and one month after treatment was completed.
**Notes**	

Risk of bias table

Bias	Authors' judgement	Support for judgement
Random sequence generation (selection bias)	Unclear risk	Patricipants were randomly assigned to one of 2 groups: active neurofeedback or control. However, no mention of random sequence generation used.
Allocation concealment (selection bias)	Unclear risk	Unclear as to whether children knew to which group they would be allocated to.
Blinding of participants and personnel (performance bias)	High risk	Both patients (children) and clinicians administering NF were aware of which group they were allocated to.
Blinding of outcome assessment (detection bias)	Low risk	All assessments were conducted by blinded, graduate-level research staff.
Incomplete outcome data (attrition bias)	Unclear risk	Of the 37 children who entered the trial, 32 completed the final assessments. Attrition rate of 13.5% (5 out of 37 dropped out).
Selective reporting (reporting bias)	High risk	Did not report on Chid Dissociative Checklist; NIH Toolbox cognitive battery, or PTSD-RI assessments.
Other bias	Low risk	No statements related to conflicts of interest were noted.

Schuurmans 2021

**Methods**	Randomized controlled trial at a residential community center in Holland.
**Participants**	Adolescents 10-18 years of age with a Children's Revised Impact of Event Scale (CRIES-13) score of greeater than or equal to 30 (with this score indicative of levels of posttraumatic symptoms. Overall, there were 46 males (59.7%); mean age of 15.25 +/- 1.79 years; mean IQ score of 86.43 +/- 1.72.
**Interventions**	Muse a game based meditation applicaiton with a brain sensing headband that utilizes neurofeedback (consisting of two 15-20 minute gameplay sessions a week for 6 consecutive weeks plus treatment as usual (TAU) (n=37) vs.TAU (medication, animal assiste-therapy, psychomoter therapy) (n=40). With Muse, when a participant's mind was calm, the environent on the computer screen

	(iPad) showed calm and settled winds but the winds picked up and blew when the participant's mind became more active.<br>Each participant was provided with a stress task of composing the end of a story 5 minutes after being exposed to the story and; presenting the end of the story in front of a camera. As part of the stress task, patients were given a song book and told they had to sing the song in front of the camera.
**Outcomes**	Basal autonomic nervious system (ANS) activiity evaluated during the following phases of the stress task during the intervention: anticipation speech task, speech task, anticipation song task and song task; Basal hypothalmic-pituitary-adrenal (HPA) axis activity - hair cortisol (hC) levels - evaluated at baseline, week 8 and week 16. Hair cortisol levels are significantly elevated in stressed individuals.<br>HPA saliva cortisol (sC) levels with signifiantly elevated in stressed individuals - obtained during each session - 20 minutes before stress task started; immediately pre-stress task initiation; immediately post-stress task completion; and; 10, 20, and 40 minutes after the stress task had ended.
**Notes**	Study registered with the Netherlands Trial Reigster under ID NL6689. Study was not powered a priori. In order to achieve a 80% power to detect a difference between conditions, the sample size should have been 95 participants.

Risk of bias table

**Bias**	**Authors' judgement**	**Support for judgement**
Random sequence generation (selection bias)	Low risk	Participants randomly allocated using python script randomization.
Allocation concealment (selection bias)	High risk	Allocation to the conditions was not masked.
Blinding of participants and personnel (performance bias)	High risk	Was not feasible to blind participants or their caregivers (mentors).
Blinding of outcome assessment (detection bias)	Unclear risk	Was not clear as to whether those assessing outcomes were blinded to treatment arms.
Incomplete outcome data (attrition bias)	Low risk	4 or 5.19% of participants dropped out of the trial.
Selective reporting (reporting bias)	Low risk	All outcomes were reported on.
Other bias	Low risk	Authors declared no competing interests. Funder had no part in the design of the study, data collection, analyses, interpretation or in the writing of the manuscript.

van der Kolk 2016

**Methods**	Prospective randomized controlled study conducted between July 1, 202 and July 1, 2015 in the US. Unclear if patients were treated at a single center or multictr.
**Participants**	52 individuals with chronic PTSD (having received six or more months of trauma focused therapy without sustained self-reported clinical improvement). Neurofeedback group (N=28); Waitlist/control group (N=24). Neurofeedback group was 46.04+/-12.89 years with 92.6/7.4 breakout female to male. Control group was 42.45+/-13.5 years of age with 77.3/22.7 breakout female to male. Both groups continued all ongoing treatments (psychotherapeutic and pharmacological). Exclusion criteria included: unstable medical condition, receiving disability benefits, active suicide risk, psychotic or bipolar disorder, traumatic brain injury, history of seizures, substance or alcohol abuse, ongoing traumatic exposure, Global Assessment of Functioning (GAF) score <40.
**Interventions**	Neurofeedback [NF] (Brain computer interaction devices/EEG) subjects had 24 training sessions, twice weekly, each lasting up to 30 minutes. NF targeted temporal lobe EEG patterns. Subjects received auditory and visual feedback indicating reward towards progress in simple computer games. Neurofeedback patients also continued with psychotherapy plus medications. Waitlist subjects: continued psychotherapy plus medications.
**Outcomes**	Primary outcome: Clinician Administered PTSD Scale (CAPS) measured at baseline, post-treatment and one month follow-up post-treatment; Secondary outcomes: Davidson Trauma Scale (DTS) measured at baseline, after week six, post-treatment and one month follow-up post treatment; Inventory of Altered Self-Capacities (IASC) measured at baseline, after week six, post-treatment and one month follow-up post treatment.
**Notes**	*Funders did not contribute to the design or execution of the study. No competing interests were noted.*

Risk of bias table

**Bias**	**Authors' judgement**	**Support for judgement**
Random sequence generation (selection bias)	Low risk	*Computer generated randomization program.*
Allocation concealment (selection bias)	Unclear risk	Unclear whether allocation concealment was undertaken to prevent knowledge of which arm patient would be allocated to.
Blinding of participants and personnel (performance bias)	High risk	Patients and clinicians were aware to which group they were treating.
Blinding of outcome assessment (detection bias)	Low risk	*Strenuous efforts were made to keep evaluators blind to treatment condition, though in one case the blinding was inadvertently compromised.*

Incomplete outcome data (attrition bias)	Unclear risk	Of the 28 subjects randomized to neurofeedback 22 completed the treatment protocol (21.4% attrition rate). Of the 24 subject randomized to waiting list, 22 completed the protocol (8.3% attrition rate). Overall attrition rate was 15.3%
Selective reporting (reporting bias)	Low risk	All outcomes identified in methods section were reported on in results section. Although adverse events were not noted as an endpoint in the methods section, they were reported on in the results section.
Other bias	Low risk	*Funders did not contribute to the design or execution of the study. No competing interests were noted.*

Winkeler 2022

**Methods**	Randomized controlled single center inpatient trial taking place in Germany from May 2019 to April 2021.
**Participants**	Patients with a diagnosed eating disorder (ICD10 50.1-50.8) and; concomitant PTSD (ICD 10 F43.1) and; with and average age of 28.4+/-5.9 years of age were entered into th trial. Patients exhibited chronic PTSD (>1 yr). 31 patients experienced sexual assault; 6 patients had experienced neglect in their childhood. All patients were female per email reply from A Winkeler on October 5, 2023.
**Interventions**	Infra-low frequency (ILF) neurofeedback (N=18); media supported relaxation (N=18). These treatments occurred twice per week over 6 weeks (12 sessions total) at 30 minutes per session. Treatment also included psychotherapy which occurred individually (75 minutes per week and as a group (150 minutes per week) over the 6 weeks as well as nutrition counseling, body awareness therapy, psycho-education and physical education.
**Outcomes**	Primary outcomes were the eating disorder examination questionnaire (EDE-Q) and the impact of event scale-revised (IES-R) [for PTSD] measured at baseline and the end of the trial. The secondary outcome assessed was the global assessment of psychological treatment success (GAPS). Complications associated with treatment were assessed over the course of the treatment.
**Notes**	Funding support provided by the Anthony and Jeanne Pritzker Foundation.

Risk of bias table

**Bias**	**Authors' judgement**	**Support for judgement**
Random sequence generation (selection bias)	Low risk	*"The randomizaton was performed in a balanced manner by using the True Random Number Generator freely available on line to generate 6 blocks of equal length permuted with regard to the treatment groups, which were arranged in random order on the randomization list."*

Allocation concealment (selection bias)	Low risk	Per email from lead author Anna Winkeler on October 5, 2023, randomization took place successively after inclusion in the study, immediately before the start of treatment.
Blinding of participants and personnel (performance bias)	High risk	Patients and clinicians knew to which group patients were assigned.
Blinding of outcome assessment (detection bias)	High risk	Per email from Anna Winkeler on October 5, 2023, there was no blinding.
Incomplete outcome data (attrition bias)	Unclear risk	Of the 36 patients entered into the trial - 28 patients completed the entire treatment regimen of 12 sessions.
Selective reporting (reporting bias)	Low risk	All outcomes as listed in the methods section were reported on in the results section.
Other bias	Low risk	None listed.

Yeganeh 2016

**Methods**	Mutlicenter prospective randomized controlled trial taking place in Iran during 2014.
**Participants**	30 male war veterans suffering from PTSD as diagnosed by posttraumatic stress disorder checklist (PCL). Average age of experimental neurofeedback group (N=15) was 48.2 years (range 43-60 years). Average age of control group (N=15) was 48.73 years (range 41-60 years).
**Interventions**	Neurofeedback (N-=15); 20 sessions each of 45 minutes duration. Control group (N=15) were treated under conventional care.
**Outcomes**	Posttraumatic stress disorder checklist (PCL).
**Notes**	Email sent to corresponding author on 09-20-23 and on 09-26-23 requesting additional information. The manuscript states twice that the paper was an RCT in the methods sections (abstract and main body of paper). However in the limitations section, it states that it states that the study was based on a non-random selection of patients. The assumption made is that the manuscript is an RCT.

Risk of bias table

Bias	Authors' judgement	Support for judgement
Random sequence generation (selection bias)	Unclear risk	Randomized but unclear as to randomization scheme. Note that in 2 places in the manuscript it states that the study was randomized (Methods section of abstract and in the Methods section of the main body of the manuscript). However in the limitations section it states that the study was based on a non-random selection of participants.

Allocation concealment (selection bias)	Unclear risk	Unclear as to whether treatment was initiated immediately upon randomization.
Blinding of participants and personnel (performance bias)	High risk	Participants and clinicians were not blind to treatment arms.
Blinding of outcome assessment (detection bias)	Unclear risk	Unclear as to whether individual assessing outcomes was blinded to treatment arms.
Incomplete outcome data (attrition bias)	Low risk	Thirty patients entered the trial and were reported on in the results section.
Selective reporting (reporting bias)	Low risk	PCL score was identified in methods section as outcome and reported on in the results section.
Other bias	Low risk	No other biases were noted.

Zhao 2023

**Methods**	Single center, randomized double blinded trial took place at Yale University, USA. No dates as to when trial took place.
**Participants**	Intervention group consisted of (N=14): Age: 40.2+/-14.27 years; 3 male/11 female; 50% on antidepressants. Yoke sham feedback (N=11): Age: 50.26+/-12.8 years; 1 male/10 female; 18% on antidepressants and 18% on anticonvulsants. Exclusion criteria included: history of psychosis or mania; pregnancy, history of drug dependence, active suicidality with past year or history of suicide attempt in past 2 years, changes in psychotropic medications with past 2 years, history of brain surgery, brain injury, epilepsy, any primary psychiatric diagnosis of a current major mood disorder, psychotic disorder, autism, mental retardation, of DSM-5 substance use disorder of mild or greater severity in the past 30 days, contraindication for MRI scan, active engagement in Cognitive behavioral therapy, enrolment in another research study
**Interventions**	Intervention: received feedback on amygdala activity in the individualized region of interest. Control group received recorded feedback from a matched participant from the active group (yoked). Both groups were provided with individualized mental strategies that might help them decrease their amygdala activity. Three sessions over a 3 week period for both intervention and control groups.
**Outcomes**	Primary outcome: Generalized linear model analysis of patient's ability to down-regulate their amygdala. Secondary outcome measures: Change over time of Clinician Administered PTSD scale (CAPS-5) captured at baseline, post-treatment and at 60 day follow up post treatment; changes in amygdala resting-state functional connectivity (RSFC).
**Notes**	One of the authors has a patent application for neurofeedback in a different modality.

Risk of bias table

Bias	Authors' judgement	Support for judgement
Random sequence generation (selection bias)	Low risk	Group assignment was based on a pseudo-random sequence generated by computer program prior to study initiation.
Allocation concealment (selection bias)	Low risk	No mention of breaking of allocation concealment by authors/investigators.
Blinding of participants and personnel (performance bias)	Unclear risk	Patient was blinded to treatment outcome. However 7 out of 11 patients in the control group guessed they received the control intervention. Clinical psychologist was blind to intervention assignment.
Blinding of outcome assessment (detection bias)	Low risk	CAPS-5 data were collected by clinical psychologists who were blind to the group allocation of the participants.
Incomplete outcome data (attrition bias)	Low risk	Of the 27 randomized patients, 2 patients dropped out prior to trial initiation (7.4% dropout rate). Of 25 patients who entered into trial (14 intervention group and 11 in sham-control) - CAPS-5 was available at all time points.
Selective reporting (reporting bias)	Low risk	All outcomes identified in the methods section were reported on in the results section.
Other bias	Low risk	Only one conflict of interest noted - one of the 14 co-authors has a patent in neurofeedback using a different modality.

Footnotes

Characteristics of excluded studies

Askovic 2020

**Reason for exclusion**	Use of neurofeedback in PTSD but was not a randomized trial.

du Bois 2021

**Reason for exclusion**	Use of Neurofeedback treating chronic PTSD but was not an RCT.

Gapen 2016

**Reason for exclusion**	RCT of NF in treating PTSD evaluating the placement of sensors on the scalp. Patients in each arm of the trial were actively treated. Found that PTSD symptoms were reduced significantly in each group with; no difference between groups in symptom reduction. Limitations section of paper states: *"conclusion is greatly limited by a lack of a control group and a lack of the ability to*

	systematically assess changes associated with NF training."

Harlé 2020

**Reason for exclusion**	RCT using prolonged exposure to a traumatic event using psychotherapy.

McDermott 2022

**Reason for exclusion**	RCT using neurofeedback on healthy subjects only.

Protopopescu 2022

**Reason for exclusion**	RCT using cognitive remediation therapy for PTSD

Shaw 2023

**Reason for exclusion**	Duplicate of Nicholson 2020.

Characteristics of ongoing studies

Roley-Roberts 2021

**Study name**	Current State of Neurofeedback for PTSD and Findings from a Pilot Waitlist Trial of Adolescents
**Methods**	Randomized controlled trial
**Participants**	Adolescents 12-18 years of age with DSM-5 PTSD
**Interventions**	Neurofeedback (n=12); Waitlist (n=12)
**Outcomes**	Unclear as to main outcome
**Starting date**	Based on abstract it is unclear as to when trial was initiated.
**Contact information**	Michelle E. Roley-Roberts, PhD Creighton University; michelleroley-roberts@creighton.edu
**Notes**	Sent emails to Professor Roley on 12/18/23 and 12/19/23; called her on 12/19/23. No reply. Abstract of study apprears in the Journal of the Amercian Academy of Child and Adolescent Psychiatry. 2020;59:10S. Abstract # 48.3
