# Supplementary material for: Systematic review and meta-analysis of neurofeedback and its effect on posttraumatic stress disorder
Source: Front Psychiatry. 2024 Mar 21;15:1323485. doi: 10.3389/fpsyt.2024.1323485 (PMC10993781; doi:10.3389/fpsyt.2024.1323485)
Supplement: Supplementary file 3 [file DataSheet_3.pdf]

Appendix 3: Consensus on the Reporting and Experimental Design of clinical and cognitive behavioral Neurofeedback studies (CRED-nf) best practices checklist 2020

	Peniston 1991	Kelson 2013	Yeganeh 2015	van der Kolk 2016	Onton 2016	Noohi 2017	Misaki 2018	Antle 2018	Bell 2019	Nicholson 2020	Rogel 2020	Fruchtman-Steinbok 2021	Leem 2021	Schuurmans 2021	Winkeler 2022	Zhao 2023	Fine 2023	Percent meeting best practice
**Domain**																		
**Pre-experiment**																		
Pre-register experimental protocol and planned analysis	-	-	-	+	+	-	-	-	-	-	-	+	+	+	+	+	+	**47.1%**
Justify sample size	-	-	+	-	-	-	-	-	+	-	+	-	+	-	+	-	+	**35.3%**
**Control groups**																		
Employ control group(s) or condition(s)	+	+	+	+	+	+	+	+	+	+	+	+	+	+	+	+	+	**100.0%**
When leveraging experimental designs where a double-blind is possible, use a double blind	-	-	-	-	+	-	+	-	-	+	-	-	-	-	-	+	-	**23.5%**
Blind those who rate the outcomes	+	-	-	+	-	-	-	-	-	-	+	+	+	-	-	+	+	**41.2%**
Examine to what extent participants and experimenters remain blind	-	-	-	-	+	-	+	-	-	+	-	-	-	-	-	+	-	**23.5%**
In clinical efficacy studies, employ a standard of care intervention group as a benchmark for improvement	+	-	+	+	-	-	-	-	-	-	+	-	+	+	+	-	+	**47.1%**
**Control measures**																		
Collect data on psychosocial factors	-	+	-	+	+	-	+	-	+	+	+	+	-	-	+	+	+	**64.7%**
Report whether participants were provided with a strategy	+	+	+	+	-	+	+	-	+	+	-	+	-	+	-	+	+	**70.6%**
Report the strategies participants used	+	+	+	+	-	+	+	-	+	+	-	+	-	+	-	+	+	**70.6%**
Report methods used for online-data processing and artifact correction	-	+	-	+	-	+	+	-	+	+	-	+	-	-	-	+	+	**52.9%**
Report condition and group effects for artifacts	-	-	-	+	-	+	+	-	+	-	-	+	-	-	-	+	-	**35.3%**
**Feedback specifications**																		
Report how the online-feature extraction was defined	-	-	+	+	+	+	+	+	+	+	-	+	+	+	+	+	+	**82.4%**
Report and justify the reinforcement schedule	-	-	-	+	-	-	-	-	-	+	-	+	-	-	-	-	+	**23.5%**
Report the feedback modality and content	+	+	+	+	+	+	+	+	+	+	+	+	+	+	+	+	+	**100.0%**
Collect and report all brain activity variable(s) and/or contrasts used for feedback, as displayed to experimental participants	-	-	-	-	+	-	+	-	+	+	-	+	-	+	+	+	+	**52.9%**
Report the hardware and software used	+	+	-	+	-	-	+	+	+	+	+	+	+	+	+	+	+	**82.4%**
**Outcome measures**																		

	Peniston 1991	Kelson 2013	Yeganeh 2015	van der Kolk 2016	Onton 2016	Noohi 2017	Misaki 2018	Antle 2018	Bell 2019	Nicholson 2020	Rogel 2020	Fruchtman-Steinbok 2021	Leem 2021	Schuurmans 2021	Winkeler 2022	Zhao 2023	Fine 2023	Percent meeting best practice
**Domain**																		
Report neurofeedback regulation success based on the feedback signal	+	+	+	+	-	+	+	+	+	+	+	+	+	+	+	+	+	**94.1%**
Plot within-session and between-session regulation blocks of feedback variable(s), as well as pre-to-post resting baselines or contrasts	-	-	-	-	-	-	+	-	+	+	-	+	-	-	+	+	-	**35.3%**
Statistically compare the experimental condition/group to the control condition(s)/group(s) (not only each group to baseline measures)	+	+	+	+	+	+	+	+	+	+	+	+	+	+	+	+	+	**100.0%**
Include measures of clinical or behavioral significance, defined a priori, and describe whether they were reached	+	+	+	+	+	+	+	+	+	+	+	+	+	+	+	+	+	**100.0%**
Run correlational analyses between regulation success and behavioral outcomes	-	+	-	-	-	-	+	-	-	+	-	+	-	-	+	+	+	**41.2%**
**Data storage**																		
Upload all materials, analysis scripts, code, and raw data used for analyses, as well as final values, to open access data depository	-	-	-	-	-	-	-	-	-	-	-	-	-	-	-	-	-	**0.0%**
**Percent criteria met by study**	**43.5%**	**47.8%**	**43.5%**	**69.6%**	**43.5%**	**43.5%**	**73.9%**	**30.4%**	**65.2%**	**73.9%**	**43.5%**	**78.3%**	**47.8%**	**52.2%**	**60.9%**	**82.6%**	**78.3%**	**57.5%**
												66.7%						
												52.6%						

Percent meeting best practice column at right - percent of those studies meeting the particular domain best practice; Percent criteria met by study row at bottom - percent of all domains being met by the particular study. Percentage of 66.7% represents the percent of all domains being met by studies published after 2020. Percentage of 52.6% represents the percent of all domains being met by studies published prior to 2021.
