# Supplementary material for: Systematic review and meta-analysis of neurofeedback and its effect on posttraumatic stress disorder
Source: Front Psychiatry. 2024 Mar 21;15:1323485. doi: 10.3389/fpsyt.2024.1323485 (PMC10993781; doi:10.3389/fpsyt.2024.1323485)
Supplement: Supplementary file 4 [file DataSheet_4.pdf]

Appendix 4: Systematic Review search strategy						
Search terms used: Neurofeedback AND Random* AND control AND PTSD AND post AND traumatic AND Stress AND disorder						
**Source:**	**Total number of hits**	**Date search**	**Obtained article**	**Disposition (RCT of NF)**	**Disposition (not NF RCT, not an RCT, or a systematic review) but obtained**	**Number of duplicates**
Cochrane CENTRAL	44	9/25/2023	A Randomized Controlled Study of Neurofeedback for Chronic PTSD. BA van der Kolk, H Hodgdon, M Gapen, R Musicaro, MK Suvak, E Hamlin, J Spinazzola PloS one, 2016, 11(12), e0166752	RCT included in analysis		
Cochrane CENTRAL		9/25/2023	A pilot study of neurofeedback for chronic ptsd Mark Gapen, A Kolk Bessel, Ed Hamlin, Laurence Hirshberg, Michael Suvak, Joseph Spinazzola Applied psychophysiol biofeedback, 2016, No-Specified | added to CENTRAL: 31 July 2016 | 2016 Issue 7		Not RCT	
Cochrane CENTRAL		9/25/2023	Effectiveness of neuro-feedback treatment with alpha/theta method on PTSD symptoms and their executing function S Noohi, AM Miraghaie, A Arabi Biomedical research (india), 2017, 28(5), 2019-2027 |	Obtained but protocol only		
Cochrane CENTRAL		9/25/2023	Neurofeedback self-regulating training in patients with Post traumatic stress disorder: a randomized controlled trial study protocol J Leem, MJ Cheong, S-H Yoon, H Kim, H-G Jo, H Lee, J Kim, HY Kim, G-W Kim, HW Kang Integrative medicine research, 2020, 9(4)	RCT included in analysis		
Cochrane CENTRAL		9/25/2023	A randomized, controlled trial of alpha-rhythm EEG neurofeedback in posttraumatic stress disorder: a preliminary investigation showing evidence of decreased PTSD symptoms and restored default mode and salience network connectivity using fMRI AA Nicholson, T Ros, M Densmore, PA Frewen, RWJ Neufeld, J Théberge, R Jetly, RA Lanius NeuroImage. Clinical, 2020, 28, 102490	RCT included in analysis		
Cochrane CENTRAL		9/25/2023	Increased top-down control of emotions during symptom provocation working memory tasks following a RCT of alpha-down neurofeedback in PTSD SB Shaw, AA Nicholson, T Ros, S Harricharan, B Terpou, M Densmore, J Theberge, P Frewen, RA Lanius NeuroImage. Clinical, 2023, 37, 103313			Duplicate of Nicholson 2020
Cochrane CENTRAL		9/25/2023	48.3 CURRENT STATE OF NEUROFEEDBACK FOR PTSD AND FINDINGS FROM A PILOT WAITLIST TRIAL OF ADOLESCENTS ME Roley-Roberts Journal of the American Academy of Child and Adolescent Psychiatry, 2020, 59(10), S340 | added to CENTRAL: 31 December 2020	RCT included in analysis		
Cochrane CENTRAL		9/25/2023	Healing the neurophysiological roots of trauma: a controlled study examining loreta z-score neurofeedback and HRV biofeedback for chronic PTSD AN Bell, D Moss, RJ Kallmeyer NeuroRegulation, 2019, 6(2), 54-70	RCT included in analysis		
Cochrane CENTRAL		9/25/2023	Amygdala electrical-finger-print (AmygEFP) NeuroFeedback guided by individually-tailored Trauma script for post-traumatic stress disorder: proof-of-concept T Fruchtman-Steinbok, JN Keynan, A Cohen, I Jaljuli, S Mermelstein, G Drori, E Routledge, M Krasnoshtein, R Playle, DEJ Linden, T Hendler NeuroImage. Clinical, 2021, 32, 102859	RCT included in analysis		

Source:	Total number of hits	Date search	Obtained article	Disposition (RCT of NF)	Disposition (not NF RCT, not an RCT, or a systematic review) but obtained	Number of duplicates
Cochrane CENTRAL		9/25/2023	Amygdala-related EEG Neuro-Feedback as an add-on Therapy for treatment-resistant Childhood Sexual Abuse PTSD: feasibility Study<br>NB Fine, L Helpman, DB Armon, G Gurevitch, G Sheppes, Z Seligman, T Hendler, M Bloch<br>Psychiatry and clinical neurosciences, 2023	RCT included in analysis		
Cochrane CENTRAL		9/25/2023	Amygdala downregulation training using fMRI neurofeedback in post-traumatic stress disorder: a randomized, double-blind trial<br>Z Zhao, O Duek, R Seidemann, C Gordon, C Walsh, E Romaker, WN Koller, M Horvath, J Awasthi, Y Wang, E O'Brien, H Fichtenholtz, M Hampson, I Harpaz-Rotem<br>Translational psychiatry, 2023, 13(1), 177	RCT included in analysis		
Cochrane CENTRAL		9/25/2023	Neural affective mechanisms associated with treatment responsiveness in veterans with PTSD and comorbid alcohol use disorder<br>KM Harlé, AN Simmons, SB Norman, AD Spadoni<br>Psychiatry research. neuroimaging, 2020, 305, 111172		Obtained but an RCT using psychotherapy	
Cochrane CENTRAL		9/25/2023	The impact of neurofeedback training on children with developmental trauma: a randomized controlled study<br>A Rogel, AM Loomis, E Hamlin, H Hodgdon, J Spinazzola, B van der Kolk<br>Psychological trauma : theory, research, practice and policy, 2020, 12(8), 918-929	RCT included in analysis		
Cochrane CENTRAL		9/25/2023	Correction: a randomized controlled study of neurofeedback for chronic PTSD (PLoS ONE (2016) 11: 12 (e0166752) DOI: 10.1371/journal.pone.0166752)<br>BA van der Kolk, H Hodgdon, M Gapen, R Musicaro, MK Suvak, E Hamlin, J Spinazzola<br>PloS one, 2019, 14(4)			Obtained but duplicate of van der Kolk 2016
Cochrane CENTRAL		9/25/2023	Evaluation of Neurofeedback for Posttraumatic Stress Disorder Related to Refugee Experiences Using Self-Report and Cognitive ERP Measures<br>M Askovic, AJ Watters, M Coello, J Aroche, AWF Harris, J Kropotov<br>Clinical EEG and neuroscience, 2020, 51(2), 79-86		Obtained but not an RCT	
Cochrane CENTRAL		9/25/2023	Rt-fMRI neurofeedback-guided cognitive reappraisal training modulates amygdala responsivity in posttraumatic stress disorder<br>J Zweerings, P Sarkheil, M Keller, M Dyck, M Klasen, B Becker, AJ Gaebler, CN Ibrahim, BI Turetsky, M Zvyagintsev, G Flatten, K Mathiak<br>NeuroImage. Clinical, 2020, 28, 102483		Obtained but not an RCT	
Cochrane CENTRAL		9/25/2023	Effectiveness, Cost-Utility, and Safety of Neurofeedback Self-Regulating Training in Patients with Post-Traumatic Stress Disorder: A Randomized Controlled Trial<br>J Leem, MJ Cheong, H Lee, E Cho, SY Lee, GW Kim, HW Kang<br>Healthcare (Basel, Switzerland), 2021, 9(10) |	RCT included in analysis		
PubMed	26	7/10/2023				9

Source:	Total number of hits	Date search	Obtained article	Disposition (RCT of NF)	Disposition (not NF RCT, not an RCT, or a systematic review) but obtained	Number of duplicates
PubMed		7/10/2023	Homeostatic normalization of alpha brain rhythms within the default-mode network and reduced symptoms in post- traumatic stress disorder following a randomized controlled trial of electroencephalogram neurofeedback.<br>Nicholson AA, Densmore M, Frewen PA, Neufeld RWJ, Theberge J, Jetly R, Lanius RA, Ros T. Brain Commun. 2023 Mar 16;5(2):fcad068. doi: 10.1093/braincomms/fcad068. eCollection 2023.			Duplicate of Nicholson 2020
PubMed		7/10/2023	The Effectiveness of Using Neurofeedback in the Treatment of Post-Traumatic Stress Disorder: A Systematic Review.<br>Panisch LS, Hai AH.<br>Trauma Violence Abuse. 2020 Jul;21(3):541-550. doi: 10.1177/1524838018781103. Epub 2018 Jun 11.		Obtained to extract additional studies; review	
PubMed		7/10/2023	Electroencephalography-based neurofeedback as treatment for post-traumatic stress disorder: A systematic review and meta-analysis.<br>Steingrimsson S, Bilonic G, Ekelund AC, Larson T, Stadig I, Svensson M, Vukovic IS, Wartenberg C, Wrede 0, Bernhardsson S. Eur Psychiatry. 2020 Jan 31;63(1):e7. doi: 10.1192/j.eurpsy.2019.7.<br>PMID: 32093790 Free PMC article.		Obtained to extract additional studies; review	
PubMed		7/10/2023	Infra-Low Frequency Neurofeedback in the Treatment of Patients With Chronic Eating Disorder and Comorbid Post- Traumatic Stress Disorder.<br>Winkeler A, Winkeler M, Imgart H.<br>Front Hum Neurosci. 2022 May 6;16:890682. doi: 10.3389/fnhum.2022.890682. eCollection 2022. PMID: 35601900 Free PMC article.	RCT included in analysis		
EBSCO	9	6/6/2023	Neurofeedback Effect on Symptoms of Posttraumatic Stress Disorder: A Systematic Review and Meta-Analysis.<br>Academic Journal<br>By: Choi, Yun-Jung; Choi, Eun-Joo; Ko, Eunjung. Applied Psychophysiology & Biofeedback. Jun2023, p1-16. DOI: 10.1007/s10484-023-09593-3. , Database: Academic Search Premier		Obtained to extract additional studies; review	4
Google: NEUROFEEDBACK FOR PTSD adolescents (first 3 pages of hits)	60	12/18/2023	Review of the evidence for neurofeedback training for children and adolescents who have experienced traumatic events. Schutz CC, Herbert J. Trauma, Violence and Abuse 2023		Obtained to extract additional studies; review	
Google: NEUROFEEDBACK FOR PTSD veterans (first 3 pages of hits)	60	9/30/2023	Yeganeh ZA, Dolatshahee B, Dogaheh ER. (2016) The effectiveness of neurofeedback training on reducing symptoms of war veterans with posttraumatic stress disorder. Prac. Clin. Psych. 4(1):17-23	RCT included in analysis		
Clincial trials.gov	11	7/15/2023	NCT03244475; Onton 2012	RCT included in analysis		3
totals	210					
**Hand searches of systematic reviews**						

Source:	Total number of hits	Date search	Obtained article	Disposition (RCT of NF)	Disposition (not NF RCT, not an RCT, or a systematic review) but obtained	Number of duplicates
Review of the evidence for neurofeedback training for children and adolecents who have experienced traumatic events. Schutz CC, Herbert J. Trauma, Violence and Abuse 2023	2	12/20/2023	Schuurmans AAT, Nijhof KS, Scholte R, Popma A, Otten R. (2021). Effectiveness of game-based meditation therapy on neurobiological stress systems in adolescents with posttraumatic symptoms: a randomized controlled trial. Stress. 24:6;1042-1049.	RCT included in analysis		
		12/20/2023	Antle A, Chesick L, Sridharan S, Cramer E. (2018). East meets west: A mobile brain computer system that helps children living in poverty learn to self regulate. Personal Ubiquit. Comput. 22(4):839-866.	RCT included in analysis		
Electroencephalography-based neurofeedback as treatment for post-traumatic stress disorder: A systematic review and meta-analysis.<br>Steingrimsson S, Bilonic G, Ekelund AC, Larson T, Stadig I, Svensson M, Vukovic IS, Wartenberg C, Wrede 0, Bernhardsson S. Eur Psychiatry. 2020 Jan 31;63(1):e7. doi: 10.1192/j.eurpsy.2019.7.<br>PMID: 32093790 Free PMC article.	2	7/12/2023	Peniston E, Kullosky P. (1991) Alpha-theta brainwave neuro-feedback for Vietnam veterans with combat-related post-traumatic stress disorder. Medical Psychotherapy 4: 47-60	RCT included in analysis		
Electroencephalography-based neurofeedback as treatment for post-traumatic stress disorder: A systematic review and meta-analysis.		7/12/2023	Kelson C. (2013) The impact of EEG biofeedback on veterans' symptoms of posttraumatic stress disorder (PTSD). Chicago, IL: The Chicago School of Professional Psychology; Doctoral dissertation.	RCT included in analysis		
The Effectiveness of Using Neurofeedback in the Treatment of Post-Traumatic Stress Disorder: A Systematic Review.<br>Panisch LS, Hai AH.<br>Trauma Violence Abuse. 2020 Jul;21(3):541-550. doi: 10.1177/1524838018781103. Epub 2018 Jun 11.	0	7/15/2023				
Neurofeedback Effect on Symptoms of Posttraumatic Stress Disorder: A Systematic Review and Meta-Analysis.<br>Academic Journal<br>By: Choi, Yun-Jung; Choi, Eun-Joo; Ko, Eunjung. Applied Psychophysiology & Biofeedback. Jun2023, p1-16. DOI: 10.1007/s10484-023-09593-3. , Database: Academic Search Premier	0	6/10/2023				
Totals hand search	4					
Totals	214			17	8	19
Disposition - not and RCT						
