# Supplementary material for: Systematic review and meta-analysis of neurofeedback and its effect on posttraumatic stress disorder
Source: Front Psychiatry. 2024 Mar 21;15:1323485. doi: 10.3389/fpsyt.2024.1323485 (PMC10993781; doi:10.3389/fpsyt.2024.1323485)
Supplement: Supplementary file 5 [file DataSheet_5.pdf]

Appendix 5: Sensitivity and statistical power - sample size required for Cohen's d effect size

Use of G Power 3.1.9.7 to calculate sample sizes based on Cohen's d

**Study**	**Sample size in trial**	**Test applied**	**Power @ 80% to detect Cohen's d effect size - number of patients required**		**Sample size based on power calc as identified in the study**	**Power used in study**	**Cohen's d used in study**	**Notes**
	**N**		**0.5**	**0.8**				
Peniston 1991	29	Chi Square	96	38	N/A			
Kelson 2013	10	Mann Whitney U	134	54	N/A			
Yeganeh 2015	30	ANCOVA	116	54	30	0.76	0.5	
van der Kolk 2016	52	ANCOVA	150	71	N/A	0.77	0.8	post hoc power
Onton 2016	72	Unclear			N/A			
Noohi 2017	30	ANCOVA	116	54	N/A			
Misaki 2018	36	ANCOVA	126	59	N/A			
Antle 2019	21	2X3 mixed ANOVA	98	46	N/A			
Bell 2019	23	nonparametric signed rank Wilcoxon and Mann Whitney U analyses	134	54	23		0.2, 0.5, 0.8	
Nicholson 2020	38	independent sample t test	128	52	N/A			
Rogel 2020	37	ANOVA	128	60	37		0.2, 0.5, 0.8	
Fruchtman-Steinbok 2021	38	one way ANOVA	129	61	N/A			
Leem 2021	19	two sample t test	128	52	19	0.9	1.1	
Schuurmans 2021	77	Chi Square	145	57	N/A			
Winkeler 2022	36	ANOVA	126	59	34	0.8	0.25	
Zhao 2023	25	independent sample t test	128	52	N/A			
Fine 2023	55	ANCOVA	154	73	55	0.8	0.16	Not Cohen's d but Eta-squared (η2); with >0.14 reflecting a large effect size
Average	36.94		127.25	56.00				
Std dev	18.01		15.78	8.41				
